# Functional metatranscriptomics of the rhizosphere: towards an understanding of metabolic processes of the microbial carbon pump

**DOI:** 10.1093/femsec/fiag097

**Published:** 2026-09-02

**Authors:** Julian Ruggaber, Sonja Wende, Sizhong Yang, Marco Keiluweit, Steffen Kolb

**Affiliations:** Leibnizzentrum für Agrarlandschaftsforschung (ZALF) e.V., 15374 Müncheberg, Germany; Thaer Institute, Faculty of Life Sciences, Humboldt University of Berlin, 10099 Berlin, Germany; Leibnizzentrum für Agrarlandschaftsforschung (ZALF) e.V., 15374 Müncheberg, Germany; Leibnizzentrum für Agrarlandschaftsforschung (ZALF) e.V., 15374 Müncheberg, Germany; University of Lausanne, Faculty of Geosciences and the Environment, 1022 Lausanne, Switzerland; Leibnizzentrum für Agrarlandschaftsforschung (ZALF) e.V., 15374 Müncheberg, Germany; Thaer Institute, Faculty of Life Sciences, Humboldt University of Berlin, 10099 Berlin, Germany

**Keywords:** SOC, soil microbial carbon pump, agricultural soil, metatranscriptome, soil erosion

## Abstract

The soil microbiome drives soil organic carbon (SOC) transformation, shaped and impacted by plant growth stage and soil management such as tillage. The soil microbial carbon pump conceptually links the degradation of plant- and microbe-derived compounds and the neosynthesis of microbial biomass, as a driver of SOC buildup. Using metatranscriptomic sequencing, we characterized actively expressed microbial functions associated with the microbial carbon pump in the *Brassica napus* rhizosphere across growth stages and under simulated erosion, using a tailored KEGG Orthology (KO) approach complemented by CAZy analysis of carbohydrate-active enzyme transcripts. A clear growth stage effect emerged with higher transcript abundances at flowering. Transcripts for substrate-binding proteins and permeases of ABC transporters (e.g. xylose, trehalose, phospholipids) increased at flowering, while plant polymer-degrading transcripts remained unaffected. Elevated transcripts for chitin synthase, *glmS* (peptidoglycan precursor), and EPS-related genes (*ExoY, algF, cysE*) at flowering suggested enhanced microbial activity. Simulated soil erosion impacted only two KO transcripts. CAZy results showed the same pattern with GH related to sugars and plant polymers increased at flowering. Despite functional shifts, taxonomic composition stayed stable for most affected transcripts. Our study uncovered metabolic pathways associated with SOC transformation and microbe-derived SOC neosynthesis, offering insights into microbial contributions to SOC formation and persistence.

## Teaser

The study experimentally studied metabolic-associated transcript expression of the soil microbial carbon pump in the rhizosphere as affected by plant growth stage and simulated soil erosion through a tailored metatranscriptome analysis. The bacterium icon used in the featured image was adapted from a design by Magnific from Flaticon.com

## Introduction

Soils are an essential component of global strategies to mitigate climate change by capturing excess atmospheric CO_2_ and increasing soil organic carbon (SOC) through improved management practices (Rumpel et al. 2020). Soil erosion is a major threat to SOC in agricultural soils but can also promote rapid SOC formation (Harden et al. 1999, Remus et al. 2018, Wang et al. 2023). Agricultural soils subjected to erosion generally benefit from enhanced fertility and water retention due to higher SOC levels (Sofo et al. 2022). Erosion-prone soils have a high potential to rapidly sequester C (carbon) and may act as temporary or long-term C sinks (Stewart et al. 2007, Georgiou et al. 2022, Wenzel et al. 2022, Begill et al. 2023).

Soil-borne bacteria and fungi are key drivers of SOC formation through the transformation of root exudates, plant litter, and SOC, which subsequently leads to the formation of microbe-derived SOC. The continuous transformation of C by microorganisms in soils is known as the Soil Microbial C Pump (SMCP) (Liang et al. 2017, Liang 2020, Wang et al. 2021). Microbe-derived SOC, mostly composed of necromass (dead cell debris), can constitute up to 50% of SOC in croplands (Angst et al. 2021, Wang et al. 2021). Root litter and exudates additionally contribute to SOC formation (Nguyen (2009), Pausch and Kuzyakov 2018, Ling et al. 2022, Kaštovská et al. 2024). About 20–30% of photosynthetically assimilated C is allocated belowground as root biomass and exudates, depending on the growth stage and crop type (Pausch and Kuzyakov 2018). Root exudation varies by crop and is particularly high in rapeseed and represents a main C input to soil throughout crop growth, with additional inputs from senescent roots in later stages (Tang et al. 2021). Although a core rhizosphere microbiome persists across growth stages, its functional activity shifts with plant development (Chaparro et al. 2013). Root exudation patterns are dynamic and influenced by both soil conditions and microbial substrate preferences within the rhizosphere microbiome (Zhalnina et al. 2018, Canarini et al. 2019).

Rhizosphere soil contributes a larger share to necromass formation than bulk soil and thus plays a major role in SOC formation (Wang et al. 2025). Senescent roots are mainly composed of cellulose and hemicellulose interlinked with pectin and lignin, making them a suitable substrate for soil microorganisms in the rhizosphere (Tian et al. 2022). These plant-derived polymers are hydrolyzed by endo- and exo-hydrolyzing enzymes like the bacterial cellulosome, extracellular and membrane-bound enzymes and fungal peroxidases which have varying substrate specificities (Artzi et al. 2017, Nuccio et al. 2020). Other prominent polymers in the rhizosphere include microbial cell wall residues, i.e., bacterial peptidoglycan (PGN) and fungal chitin and melanin, highlighting microbial anabolism as a central component of the complex network leading to microbe-derived SOC formation (Liang et al. 2017, Angst et al. 2021, Wang et al. 2021, Camenzind et al. 2023). PGN and its precursors are synthesized by a wide range of enzymes, many of which are also involved in other basic cellular functions (Garde et al. 2021). PGN decomposition is likewise catalyzed by numerous enzymes of which N-acetylmuramoyl-L-alanine amidase and lysozyme are specific to PGN degradation (Vollmer et al. 2008, Humann and Lenz 2009). Chitin, the most abundant natural amino polysaccharide, is the main component of fungal cell walls and is synthesized by various chitin synthases (W. Li et al. 2016, Chen et al. 2022) and degraded by diverse microbial chitinases (Rathore and Gupta 2015, Li et al. 2016, Kandeler 2024).

Root exudates consist mainly of amino acids, sugars, organic acids, mucilage polymers, and low-molecular-weight secondary plant compounds, all of which are suitable substrates for rhizosphere microorganisms (Chaparro et al. 2013, Zhalnina et al. 2018, Vives-Peris et al. 2020). Mono- and oligosaccharides exuded by roots or through microbial hydrolysis of polymers are transported into microbial cells via substrate-specific ATP-binding cassette (ABC) transporters. These transporters consist of a substrate-binding (S-B) protein at the extracellular membrane, a permease that mediates molecular passage and an ATP-binding protein in the cytoplasm (Wilkens 2015, Thomas and Tampé 2020). Fungi also possess ABC transporters, which act mostly in the export of molecules, whereas uptake is mostly mediated by the less substrate-specific major facilitator superfamily (Nogueira et al. 2020).

The SMCP concept emphasizes the interplay between microbial anabolism and catabolism. However, these processes are tightly linked to motility and substrate uptake of soil microorganisms. Bacteria often actively move through the soil matrix toward nutrient-rich zones via flagella-driven chemotaxis, which can be observed particularly in the rhizosphere and mycosphere (Scharf et al. 2016, Begum et al. 2019, Colin et al. 2021). Most bacteria inhabit mineral and organic surfaces within biofilms composed of extracellular polymeric substances (EPS), which facilitate adhesion, protection, and nutrient retention (Flemming and Wingender 2010, Costa et al. 2018, Oliva et al. 2024, Oliva et al. 2025). Fungi, by contrast, explore their environment through the growth of hyphae that extend toward nutrient- and water-rich microsites (Begum et al. 2019). Furthermore, the interplay between polymer degradation by extracellular enzymes and the subsequent uptake of monomers such as sugars, fatty acids, and other labile compounds is regulated by membrane transport systems that respond dynamically to rhizosphere conditions (Zeng and Charkowski 2021). Quantifying the importance of these interconnected anabolic and catabolic processes in soil–plant systems is essential for a mechanistic understanding of the SMCP.

Messenger RNA (mRNA)-based analyses can directly identify actively transcribed genes encoding enzymes responsible for specific metabolic processes (Kandeler 2024). Despite the challenges posed by the instability and low abundance of mRNA relative to ribosomal RNA (rRNA) and DNA, this approach offers the advantage of capturing specific microbial activity without interference from relic nucleic acids (Kozjek et al. 2023, Karaoz et al. 2024). Metatranscriptomics enables the detection of the complete set of genes transcribed by the soil microbiome at the time of sampling (Carvalhais et al. 2012). Depletion of rRNA or poly(A) mRNA enrichment can be applied to increase the proportion of mRNA reads in datasets targeting functional aspects of the metatranscriptome (Karaoz et al. 2024; Täumer et al. 2022). Additionally, the choice of reference databases used for transcript identification strongly influences results. Databases such as Carbohydrate-Active enZYmes database (CAZy), KEGG Orthology (KO), or more specific ones (antiSMASH, LCdb) cover many, but not all relevant genes (Kanehisa 2002, Cantarel et al. 2009, Blin et al. 2024). Hence, a tailored analytical approach adapted to the research question is essential for elucidating specific metabolic activities in the rhizosphere microbiome.

We conducted a pot experiment with oilseed rape (*Brassica napus* L.) grown in an agricultural soil to investigate which functional transcripts related to the SMCP are present in the rhizosphere and how they are affected by growth stage and simulated tillage erosion through subsoil admixture. We used a tailored KO-based approach with a priori definition of genes relevant for the SMCP and additionally used CAZy to investigate carbohydrate-active enzymes. We also aimed to explore which KOs may serve as future markers for the SMCP and whether the taxonomic composition of the functional transcripts changed.

We hypothesized that (i) the absolute transcript abundances related to the SMCP increase in response to root exudation and the availability of plant polymers at the later growth stage whereas relative transcript abundance remains unchanged. (ii) Simulated erosion leads to quantitative reduction in transcript abundance and has a modulatory effect on the relative abundance of selected transcripts. (iii) CAZy transcripts affected by experimental factors mainly belong to glycoside hydrolases. (iv) Pronounced changes in taxonomic composition can be observed in KOs affected by experimental factors as selected microorganisms grow in response to the available substrate.

## Material and methods

### Site description

The soil used in the experiment originated from the Carbo-D-ZALF research site in Dedelow, Northeast Germany (N53.379, E 13.785) (Sommer et al. 2016). The landscape is characterized by hummocky moraines, and kettle hole catchments used for agriculture, with the investigated soil classified as a Nudiargic Luvisol (FAO) from a mid-slope position with an Ap-Bt-Ck horizon sequence (van der Meij et al. 2017). The topsoil (0-30 cm) consists of an Ap horizon (61% sand, 26% silt, 13% clay) with 0.86% SOC, followed by a Bt horizon (30-60 cm) with 0.16% SOC (54% sand, 27% silt, 19% clay). Soil characteristics and nutrient status were measured in the central laboratory at the ZALF headquarters (Table S1). The Ap and Bt horizons were excavated from a soil profile at the Carbo-D-ZALF research site and transferred to ZALF headquarters, where the soil was air-dried before the experiment.

### Experimental design and plant cultivation

**Figure 1:**
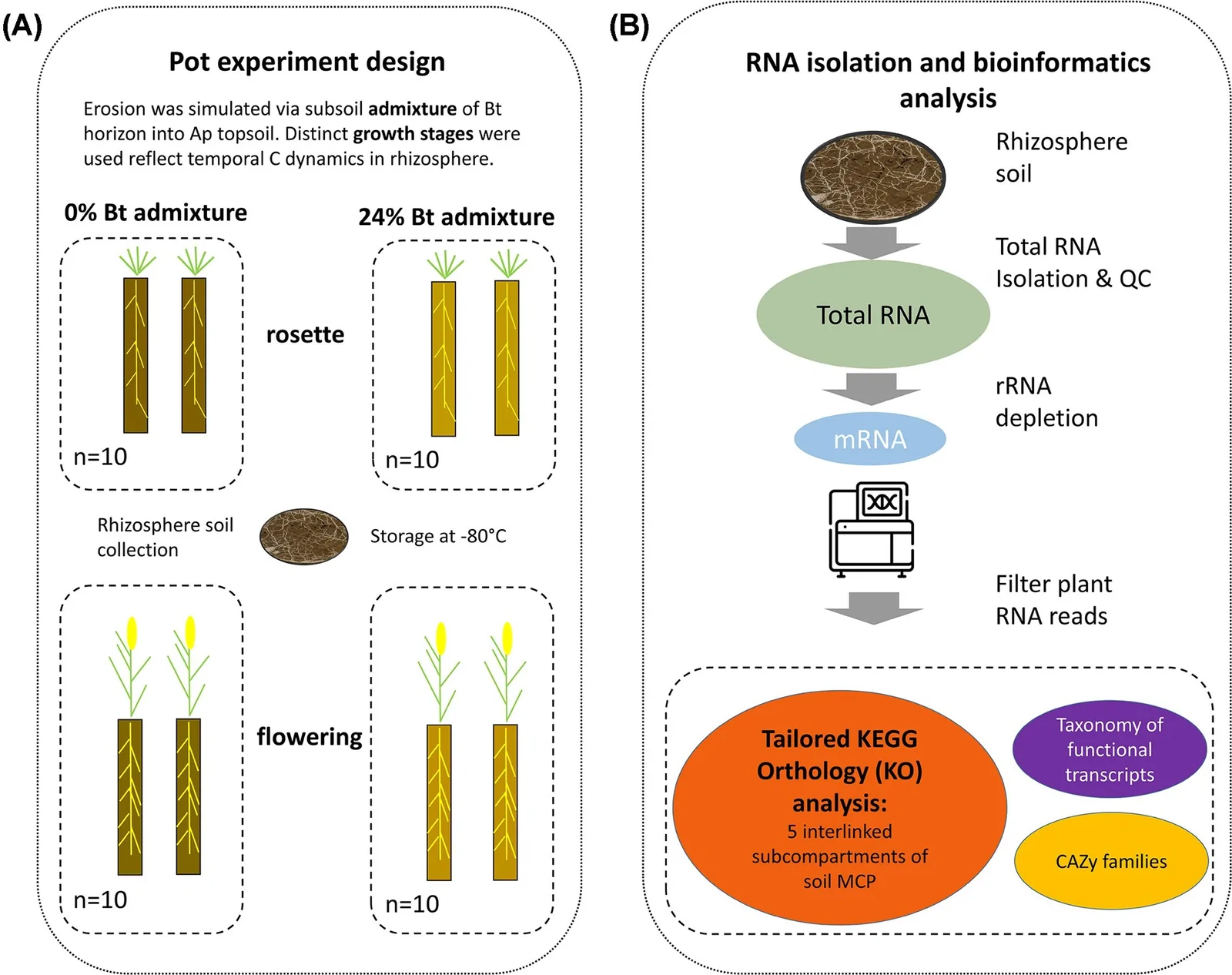
(A) Setup of the pot experiment with a subsoil admixture treatment to simulate soil erosion and plant growth stages (rosette vs flowering) to obtain rhizosphere soil.(B) RNA extraction and processing in the laboratory followed by bioinformatic analysis with the tailored approach based on KEGG Orthology (KO), Carbohydrate-Active Enzymes (CAZy) and taxonomic analysis of selected functional transcripts. Bt, subsoil horizon. QC, quality control. SMCP, soil microbial carbon pump.

The air-dried soil was sieved (5 mm mesh size) before the soil was rewetted in sterile freezing bags with 150 ml of deionized water. Twenty-two polypropylene pots (104.6 mm inner diameter, 35 cm height) were prepared for each soil treatment and contained 3513 g dry weight (dw) soil per pot. The 0% subsoil admixture treatment without simulated erosion contained 3513 g of dw soil of the Ap horizon without Bt addition. The 24% subsoil admixture treatment, simulating tillage erosion, contained 2670 g of dw soil of the Ap horizon and 843 g of dw soil of the Bt subsoil horizon. The 24% treatment was thoroughly mixed before fertilization to ensure homogenization of the soil horizons (Fig. 1). Tillage erosion is the dominant form of erosion on the research site, leading to the admixture of Bt subsoil by tillage operations into the topsoil (Sommer et al. 2016). Basal fertilization consisted of 1 g of Wopil (Siegfried W. Arnold e.K., Halle, Germany) (14% N (NH_4_ and NO_3_), 6% P (P_2_O_5_), 24% K (K_2_O) 3% Mg (MgO) and trace elements (B, Fe, Cu, Mn, Mo, Zn) and 0.1 g Kieserit (25% MgO, 20% SO_3_) dissolved in 100 ml of H_2_O per pot. The rewetted soil was transferred to the pots (104.6 mm inner diameter) and filled without compaction to a height of 31.5 cm to reach a bulk density of 1.3 g cm^-3^. The remaining water to reach 60% water-holding capacity (WHC) was then added. Three presoaked, uncoated seeds of oilseed rape (*Brassica napus* L. (cultivar CAMPINO, NPZ KG)) were placed 15 mm deep into the soil, the top 5 cm of the soil was not fertilized to ensure seedling emergence. After two weeks, a single plant was selected in each pot, and the other seedlings were removed.

The plants were cultivated in growth chambers under simulated spring to early summer conditions representative of the research site. Pots were randomly placed in four rows in the growth chamber. During the first five weeks, the plants were kept at 9 °C during the light period and 6 °C during the dark period, with 65% rel. humidity and 12 h of light with a light intensity of 350 µmol m^−2^ s^−1^ on top of the pots. The floor of the growth chamber was regularly adjusted according to plant height to ensure approximately the same light intensity. Five weeks after planting, the temperature was increased to 15 °C during the light phase (12 °C during the dark period) while all the other conditions were kept the same. Fifty days after seed planting, the first 20 pots (10 per soil treatment) were harvested at the rosette growth stage. The remaining pots were fertilized with 1.5 times the basal fertilization described above, dissolved in 100 ml water, to maintain plant growth. Seventy days after seed planting, 20 pots (10 per soil treatment) were harvested at the flowering growth stage (Fig. 1).

### Sample collection and storage

Before sampling, plants were not watered for two days to reach about 30% WHC, which was found to be optimal for rhizosphere collection in previous experiments (Ruggaber et al. 2024). Shoots were removed and dried at 65 °C for 72 h before dry mass determination. The pots were opened at the bottom, and the soil was extracted by tapping the pots. The main root system, together with attached aggregates and soil, was removed by hand while shaking the roots vigorously. Remaining roots with attached rhizosphere soil were collected by hand for 10 min per pot, with only some fine roots remaining in the soil. Soil attached to the collected roots was defined as rhizosphere soil. Roots were then removed, and rhizosphere soil was homogenized by sieving (2 mm mesh). An aliquot of ∼1 g (± 0.0001 g) was used for dry mass determination of the rhizosphere samples. Roots were washed and dried at 65 °C for 72 h for root dry mass determination. For nucleic acid extraction five aliquots of ∼ 1.5 g of soil were shock-frozen in liquid nitrogen before storage at -80 °C. The remaining soil was stored immediately at -20 °C. Bulk soil was collected after removal of the roots. The soil was mixed thoroughly before about 300 g of fresh soil was sieved (2 mm) and used for further analysis. About 30 g of soil was used for dry mass determination; the remaining soil was stored as mentioned above. Microbial biomass C (C_mic_) was determined in samples stored at -20 °C with the chloroform fumigation method (Brookes et al. 1985) with 5 g of soil and 20 ml of 0.05 M K_2_SO_4_ for extraction while horizontally shaking for 30 min. Measurement of C content was performed with a multi N/C 3100 (Analytik Jena, Germany) and calculated with the 0.45 correction factor described by Vance et al. (1987). All equipment for sampling was sterilized thoroughly between samples with 70% EtOH, glass beakers used for intermediate sample storage were covered with aluminum foil and sterilized in the oven at 200 °C for 60 min before the sampling campaign.

### Isolation of RNA from rhizosphere soil

RNA was extracted from ∼1.5 g of soil with the RNeasy PowerSoil Total RNA Kit (Qiagen, Cat. no. 12866–25) with ROTI®Phenol/Chloroform/Isoamylalkohol (25 : 24 : 1, pH 7.5–8.0) supplied by Carl Roth Scientific. Pre-experiments were conducted to optimize the RNA yield and quality according to manufacture's instructions. After RNA elution, extracts were immediately measured for RNA concentration and the A260/A280 and A260/A230 ratios using a NanoDrop ND-1000 spectrophotometer (Thermo Scientific). RNA extracts were cleaned with the RNA Clean & Concentrator-5 kit (Zymo Research) according to the manufacture's instructions, including an in-column DNase treatment. After purification RNA concentration was again measured with NanoDrop and RNA integrity number (RIN) was measured with an Agilent 2100 Bioanalyzer, using the Agilent RNA 6000 Nano Kit Guide (Agilent Technologies). A RIN value of 6.5 was considered acceptable. Absence of DNA in the samples was confirmed by running a standardized 25-cycle 16S rRNA gene PCR using the 515F-806R primer pair (Apprill et al. 2015, Parada et al. 2016) and checking the product on a 1.3% agarose gel. The purified RNA was stored at -80 °C before shipment on dry ice to Eurofins Genomics (Konstanz, Germany), where further processing of the samples was performed. Bacterial rRNA from the samples was depleted with the QIAseq FastSelect –5S/16S/23S Kit (Qiagen, Cat. no. 335 925), followed by random cDNA synthesis and library preparation. Paired-end sequencing was performed using an Illumina NovaSeq 6000 (2×150 bp) aiming for at least 45 million read pairs per sample.

### Bioinformatic processing and taxonomic annotation of non-assembled reads

Raw reads of the samples are available in the NCBI’s SRA under BioProject ID PRJNA1140101. A schematic outline of the bioinformatic workflow can be found in the supplementary materials (Fig. S1). Read quality was checked with FastQC v0.11.9. fastp v0.20.1 was used for adapter removal and read quality control (Chen 2023). Reads were trimmed when the mean quality in a sliding window of length 4 dropped below 24 and only reads with a minimum length of > 50 bp were retained. Afterwards, RiboDetector v0.2.7 (Deng et al. 2022) was used to identify and remove remaining rRNA reads. We performed taxonomic annotation of the non-assembled reads after rRNA removal to determine the origins of mRNA reads independently of the assembly process. The forward and reverse reads were merged and aligned against the NCBI non-redundant (nr) protein database (retrieved 05/2023) using DIAMOND BLASTX v2.1.6 (Buchfink et al. 2021). Alignments were taxonomically classified with MEGAN6 (Huson et al. 2016) with minSupport set to 1 and minScore set to 70. Taxonomic assignment of the non-assembled reads was used for a kingdom- and phylum-level classification.

### Assembly of contigs and annotation

Trinity v2.11.0 (Grabherr et al. 2011) was used to assemble non-rRNA reads into contigs with a minimum contig length of 160 bp for each biological replicate. Transcripts were quantified using the “align_and_estimate_abundance.pl” command from Trinity with RSEM as the estimation method (https://deweylab.github.io/RSEM) and alignment method Bowtie2 (http://github.com/benlangmead/bowtie2). This RSEM step generated the transcript-level counts and Transcripts Per Million (TPM) values. The output of the assembly was evaluated with QUAST (Gurevich et al. 2013) and BBmap (Bushnell 2014), the related summary information (N50 and BBmap) is provided in the supplementary data (Appendix B). In addition, BUSCO was used with the bacteria_odb10, fungi_odb10 and archaea_odb10 to assess the completeness and recovery of highly expressed orthologs in the transcriptome of the respective microbial groups (Tegenfeldt et al. 2025) (Appendix B).

Based on the assembled contigs we performed gene prediction using prodigal v2.6.3 (Hyatt et al. 2010) using the meta flag method. Taxonomic assignments of functionally annotated genes were obtained by aligning the corresponding predicted protein sequences against the NCBI nr database using DIAMOND blastp, followed by lowest common ancestor (LCA) classification using MEGAN (database v2.1.6) (Huson et al. 2016). The predicted genes were annotated against two functional databases with the same general workflow, with differences restricted to the functional annotation database and annotation software used.

CAZy functional annotation was performed using dbCAN v4.14, the standalone version of the dbCAN3 annotation tool, with the HMMER-based hmmsearch_dbCAN workflow (Zheng et al. 2023). Protein sequences predicted from the Trinity transcriptome assembly were searched against the dbCAN HMM database. HMMER results were processed using a custom Python script, retaining robust hits (E-value < 1 × 10⁻¹⁰, HMM coverage ≥ 30%). Where multiple CAZy hits were obtained for the same predicted protein sequence, only the hit with the lowest E-value was retained (https://github.com/j-ruggaber-zalf/Metatranscriptome_aggregation_scripts). CAZy annotations were subsequently matched to the corresponding Trinity identifiers before adding the abundance estimates generated by RSEM and the MEGAN taxonomy. The resulting tables containing CAZy family, abundance and taxonomic annotation was used for downstream analysis.

KEGG Orthology (KO) annotation was performed using KofamScan with E-value threshold 1 ×10^−4^ (KO profile database accessed on 26.01.2024; https://github.com/takaram/kofam_scan). KofamScan results were processed using a custom Python script and further filtered using an E-value < 1 × 10⁻¹⁰. Where multiple KO hits were obtained for the same predicted protein sequence, only the hit with the lowest E-value was retained (https://github.com/j-ruggaber-zalf/Metatranscriptome_aggregation_scripts). KO annotation was subsequently matched to the corresponding Trinity identifiers before adding the abundance estimates generated by RSEM and the MEGAN taxonomy. The resulting tables containing KO, abundance and taxonomic annotation were used for downstream analysis.

Taxonomic filtering of functional transcripts identified as plant (i.e. from *Brassica napus* roots) was done by removing *Streptophyta* from the aggregated tables. For functional analysis TPM and count tables were aggregated by CAZy family or KO ID, respectively. For functional analysis of KOs, the taxonomic information was retained and the taxonomic distribution of transcripts assigned to individual KO categories was assessed.

To estimate the absolute abundance of functional transcripts, aggregated KO and CAZy transcript count matrices were used to calculate transcripts g^−1^ dw rhizosphere soil abundance matrices. The calculation of mRNA transcripts g^−1^dw soil was performed as described by Söllinger et al. (2018), and applied to soil by Täumer et al. (2022). The formula is provided in the Supplementary Method S1 and the additional data for the calculations are available in Appendix A.

### Tailored KO analysis

The a priori defined KOs relevant for the soil microbial C pump were filtered and classified into five different functional categories: (i) catabolic enzymes that catalyse polymer degradation (i.e. cellulose, chitin), (ii) anabolic enzymes that catalyse bacterial peptidoglycan and fungal chitin synthesis, (iii) cellular transporter, i.e. ABC transporters, that are responsible for substrate binding, transmembrane passage (permease) and ATP binding, (iv) extracellular polymeric substances (EPS)-producing enzymes of bacteria, and (v) chemotaxis genes of bacteria. The full list of KOs used in the tailored gene analysis is available in Appendix A.

### Statistical analysis

Statistical analyses were performed in R version 4.2.1 (2022–06–23 ucrt) (R Core Team 2022). Differences in plant growth parameters, C_mic_ and RNA extracted from rhizosphere soil were tested with a two-way ANOVA followed by the Tukey HSD test (stats package).

Following aggregation to KO or CAZy functional categories, features detected in fewer than 25% of samples across all treatments were removed prior to downstream statistical analyses to reduce noise. Estimated absolute transcript abundances and TPM values were Hellinger-transformed, and Euclidean distances were calculated using the vegan package. PERMANOVA was performed with adonis2() using 9 999 permutations to test the effects of growth stage, subsoil admixture, and their interaction. Homogeneity of multivariate dispersion was assessed with betadisper(). Principal coordinate analyses (PCoA) were calculated from the same Euclidean distance matrices using cmdscale(), and the first two axes were visualized with ggplot2.

Both CAZy and KO tailored analyses were performed with transcripts g^-1^ dw soil and TPM-normalized transcript abundances. Subsequently, data was log2(TPM+1) and log2(transcripts *g dw soil +1) respectively. Log2 transformed transcripts were analyzed using two-way analysis of variance (ANOVA) (aov() function) with growth stage and admixture as factors. The Shapiro-Wilk test was used to test for normality of residuals and Levene’s test to check for homogeneity of variance. Benjamini-Hochberg false discovery rate (FDR) p-value correction was used for p-value adjustment due to multiple hypothesis testing.

For the taxonomic analysis of selected KOs, transcript-level TPM values were retained for individual KO-annotated sequences together with their corresponding taxonomic assignments, rather than being aggregated by KO. These data were used to generate phyloseq objects (McMurdie and Holmes 2013), and taxonomy was subsequently collapsed at the phylum level. ANOVA was performed for each taxonomic group using log2(TPM+1) transformation and FDR p-value correction. Visualization of the plots was done using the ggplot2 package (Wickham 2016).

## Results

### Plant, root and shoot biomass dynamics of *Brassica napus* L.

Root biomass increased significantly from approximately 1 g dw at rosette to 2 g dw at flowering (p < 0.05), while no subsoil admixture effect occurred (Table 1). The shoot biomass tripled from the early to the latter growth stage. While the shoot biomass was lower for 24% admixture in both growth stages, this effect was only significant at flowering. The root-to-shoot ratio decreased from 0.24 to 0.17 in the 0% admixture treatment and from 0.27 to 0.2 in the 24% admixture treatment. A significant decrease in the root-to-shoot ratio was correlated with ongoing plant growth, but no erosion-dependent effect occurred. C_mic_ was significantly lower in the 24% subsoil admixture at the flowering growth stage (p<0.01), but not at the rosette growth stage. Total RNA extracted (ng g^−1^ dw soil) was highest for 0% admixture at flowering compared to all other treatments (p < 0.05).

**Table 1:** Effect of growth stage and simulated erosion by subsoil admixture on the mean dry weight (dw) of harvested *Brassica napus* shoot and root material, root-to-shoot ratio, amount of rhizosphere soil, and RNA concentration in the rhizosphere soil.

growth stage	admixture	shoot (g dw)	root (g dw)	root:shoot ratio	rhizosphere soil (g dw)	Cmic µg g^-1^ dw soil	RNA ng g^-1^ dw soil
rosette	0%	4.61 ± 0.43 c	1.11 ± 0.15 b	0.24 ± 0.03 a	43.7 ± 13.6 b	356.4 ± 162 ab	2990.8 ± 1204 b
	24%	3.79 ± 0.42 c	1.04 ± 0.16 b	0.27 ± 0.02 a	47.9 ±15.4 b	291. ± 127 ab	2300.8 ± 839 b
flowering	0%	12.26 ± 1.07 a	2.12 ±0.42 a	0.17 ± 0.04 b	65.4 ± 9.3 a	456.1 ± 195 a	4605.8 ± 1325 a
	24%	10.61 ± 0.87 b	2.09 ± 0.60 a	0.20 ± 0.05 b	66.2 ± 15.9 a	213.5 ± 85 b	2868.6 ± 1224b

*Notes*: Significant differences were determined with two-way ANOVA (*P*<0.05) followed by HSD-Tukey test, letters indicate grouping. Standard error is shown behind the mean values.

### Taxonomic identification of unassembled reads

The non-assembled reads were used for a taxonomic mapping independent of the assembly and annotation of contigs. Across all treatments, ∼81.7% of reads were affiliated with Bacteria, 4.5% with Archaea and 3.1% with Fungi, while another 0.8% of the reads were assigned to viral RNA and 0.5% to Amoebozoa and 9.4% remained unassigned (Fig. S2A). Bacteria, the most abundant group detected in the unassembled reads, was mainly composed of *Actinomycetota* (∼50%), *Pseudomonadota (∼18%), Acidobacteriota (5%), Chloroflexota* (5%) and *Nitrospirae* (∼2.5%) (Fig. S2B). Almost 90% of Archaea were assigned to *Thermoproteota*, while some *Euryarchaeota* were found alonmg with some unclassified archaeal reads (Fig. S2C). Fungi were mainly composed of *Ascomycota* (40–45%) and *Mucoromycota* (35–40%) with some *Basidiomycota* and *Chytridiomycota* (less than 10%) (Fig. S2D).

### CAZy analysis of rhizosphere metatranscriptome

Analysis of carbohydrate-active enzyme (CAZy)-annotated transcripts atCAZy level 1 revealed that glycoside hydrolases (GH) was the most abundant family, comprising around 35% of CAZy-annotated transcripts followed by glycosyltransferases (GT) which accounted for around 25% of transcripts (Fig. S3A). Approximately 20% of reads were annotated to auxiliary activities (AA), 5% to carbohydrate-binding modules (CBM), with a minor proportion belonging to polysaccharide lyases (PL). Differences between treatments were only moderate, however permutational multivariate analysis of variance (PERMANOVA) identified a significant effect of growth stage on CAZy level 2 families accounting for nearly 6% of the observed variation, and no effect of admixture or the interaction of both factors (Table S2A). Principal coordinates analysis (PCoA) demonstrated a weak separation with no clear clustering of the growth stages or admixture treatments (Fig. S4A). The most abundant CAZy level 2 family was GH13, accounting for 15% of all reads, followed by AA1, GT2, GH81 and GT4 and a substantial share of CBM48 (Fig. S3B).

**Figure 2:**
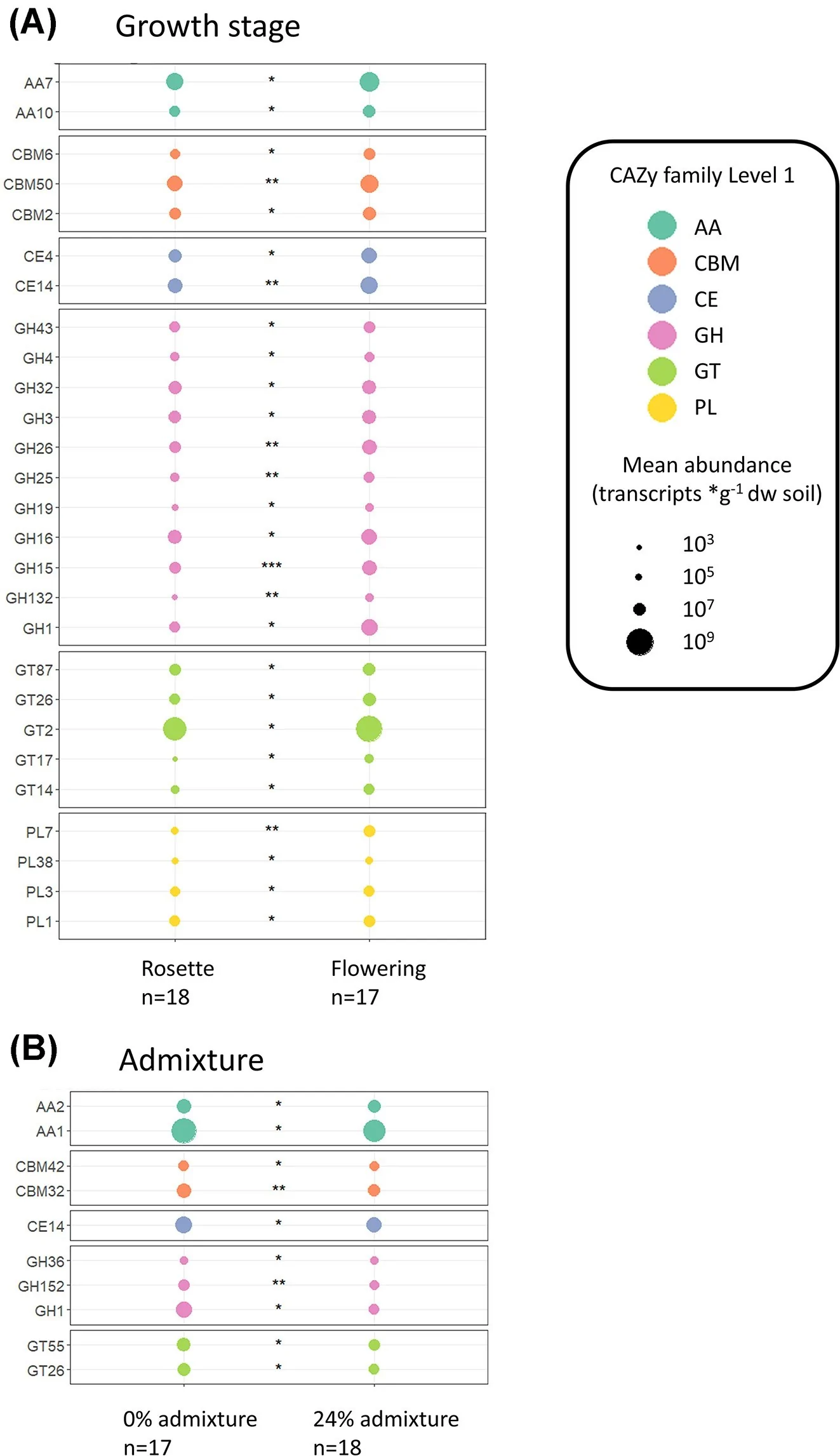
Significantly affected CAZy-annotated transcripts at level 2 by the experimental factors (A) growth stage or (B) subsoil admixture. Bubble size indicates untransformed mean values for each experimental factor, expressed as transcripts g^−1^ dw soil. Abundances were log2(x +1) transformed before two-way ANOVA with FDR *P*-value correction (* *P* < 0.05, ** *P* < 0.01, *** *P*< 0.001).

A total of 196 CAZy level 2 families were used for two-way ANOVA of which 27 were affected by growth stage and 10 by admixture treatment when CAZy annotated transcripts were calculated as transcripts g^-1^ dw soil (Figure 2). The general pattern was that the abundances were higher at the flowering growth stage and higher at the 0% admixture treatment.

Two AA families (AA7 and AA10) were increased at flowering, likewise, the carbohydrate esterases (CE) CE4 and CE14 were higher at flowering. The latter is related to chitin and N-acetyl glucosamine degradation. The CBM6, CBM50 and CBM2 were all increased, with CBM50 acting as binding module for chitin and PGN cleaving enzymes. GT87, GT26, GT17, GT14 and GT2 were all increased at flowering, with GT2 being one of the the most abundant families, encompassing a large number of enzymes for sugar and amino sugar synthesis. PL1, PL3, PL38 and PL7 were likewise increased at flowering. The latter family encompasses alginate lyases and thus is related to EPS. The largest number of CAZy-annotated families was affected in the GH family with 11 families increased at flowering. Among the most affected were GH25 peptidoglycan endo-β-1,4-N-acetylmuramidase, GH26 cellulose and xyloglucan degradation, GH15 glucosidases and GH132 glucan exo-β-1,3-glucosidase.in the text this was 2021.

Admixture led to a decrease of two CAZy-annotated transcripts in the 24% treatment AA of which the AA1, one of the most abundant CAZy annotated transcripts in the dataset, was also affected. CE14, GT55 and GT26 were also decreased at the 24% treatment. Among the three GH that were affected GH152 glucan endo-β-1,3-glucosidase showed a strong effect, with GH36 and GH1 also decreased in the 24% admixture treatment. Two CBM families decreased at 24% admixture, CBM42 and CBM32, the latter is responsible for binding of N-acetylglucosaminidase.

For TPM-normalized CAZy-annotated transcripts only four were affected by growth stage. CBM35 was increased at flowering, likewise, PL7 was increased at flowering. Furthermore, GH15 and GH132 were increased at flowering. The only transcript affected by admixture was AA1, which was decreased at 24% admixture (Appendix A).

### Tailored KEGG Orthologs (KO) analysis based on absolute reads

The tailored analysis of the KO-annotated transcripts comprised 298 a priori KOs, of which 132 were detected in the metatranscriptome and categorized into five functional categories (Table 2, Appendix A). PERMANOVA results for all KO-annotated transcripts indicated that for absolute transcripts of all KOs, growth stage explained 13% of the differences (p < 0.001), while admixture accounted for around 5% of variation and interaction had no significant impact (Tables S2B). These findings were corroborated by the PCoA showing a clear separation between growth stages and a weak admixture effect (Fig. S4B).

**Table 2:** Number of KO-annotated transcripts that were found versus the total number of KOs in the tailored metatranscriptome analysis for each functional group.

Functional groups of enzymes	Number of KOs found per total # in metatransciptome	Significantly affected transcripts (transcripts g^−1^ dw soil)		Significantly affected transcripts (TPM)	
		growth stage	subsoil admixture	growth stage	subsoil admixture
ABC Transporter	35/ 68	5	1^a^	-	-
Anabolic	15/20	2	1	1	1
Catabolic	44/111	2	1^a^	-	-
EPS	22/73	3	-	1	-
Chemotaxis	16/26	1	-	-	-
**Total**	**132/298**	**13**	**2**	**2**	**1**

*Notes*: The number of KO-annotated transcripts that were significantly affected by two-way ANOVA with FDR p-value correction, *P* < 0.05) by either subsoil admixture or growth stage are shown. Transcripts were calculated as transcripts * g^−1^ dw soil and transcripts per million (TPM). EPS = Extracellular Polymeric Substance. ^a^ bifunctional transporter protein of ribose and bacterial chemotaxis.

The tailored KO approach led to a reduction of the dataset and subsequently a weaker effect of growth stage (7%) and no significant effect of admixture was observed in the PERMANOVA (Table S2C). Likewise, PCoA showed less separation of samples compared to all KOs, with some clustering by growth stage (Fig. S4C).

**Figure 3:**
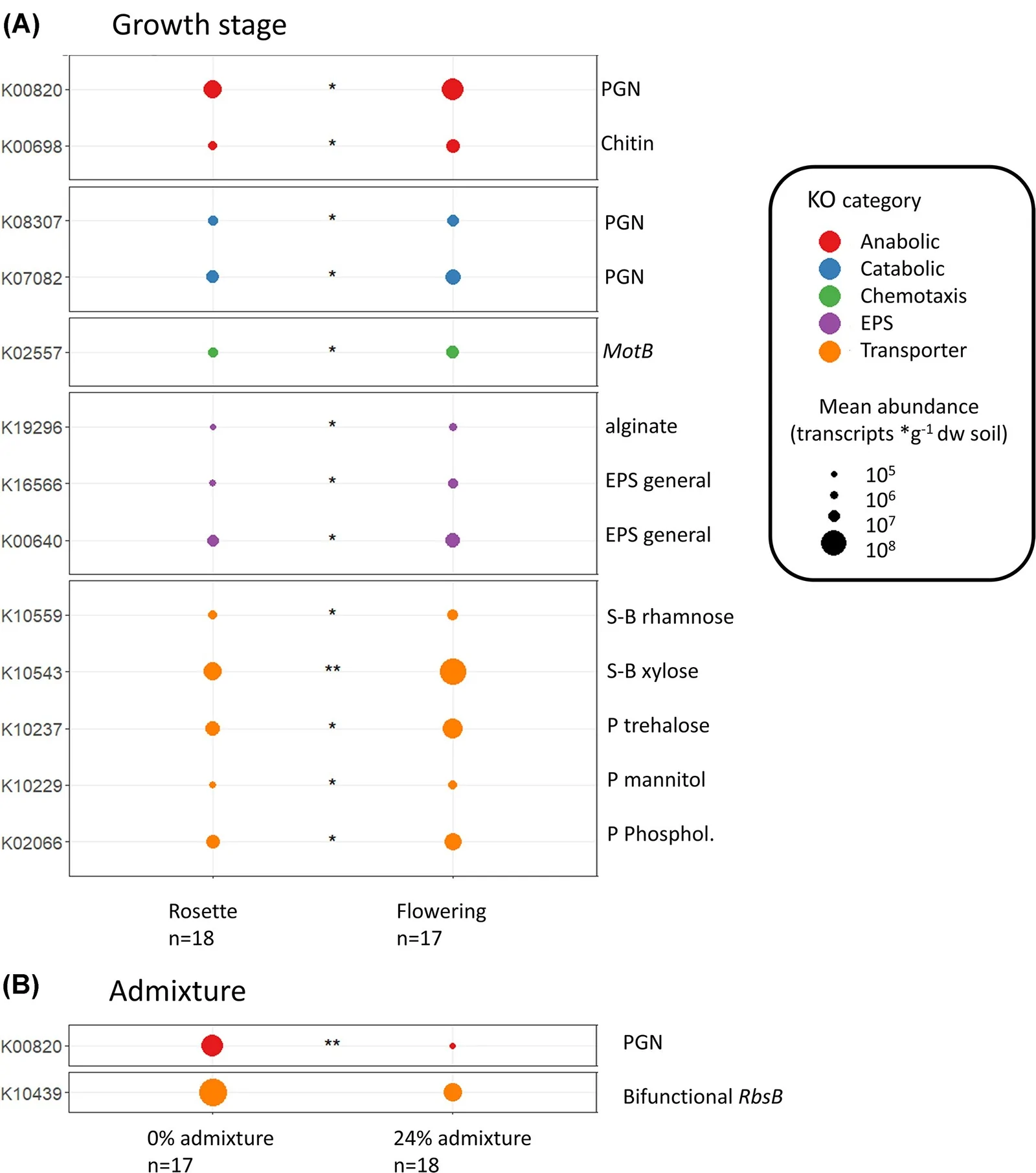
Significantly affected KO-annotated transcripts of the tailored KO analysis by the experimental factors (A) growth stage or(B) admixture. Bubble size indicates untransformed mean values for each experimental factor, expressed as transcripts g^−1^ dw soil. Abundances were log2(x +1) transformed before two-way ANOVA with FDR p-value correction (* *P* < 0.05, ** *P* < 0.01). The substrate or function of the respective KO is indicated on the right side of the graph. PGN, peptidoglycan. EPS, extracellular polymeric substance. S-B, substrate-binding protein. P, permease.

Two-way ANOVA was used to determine which transcripts of the tailored KO dataset were affected by growth stage or admixture treatment (Fig. 3). Functional analysis of KO-annotated transcripts based on absolute transcript abundance (transcripts g⁻¹ dw soil) revealed distinct shifts in both anabolic and catabolic processes at the flowering stage. Among the anabolic transcripts, chitin synthase (K00698) exhibited the strongest upregulation, suggesting enhanced fungal cell wall synthesis at flowering (Fig. S5A). Additionally, the glutamine-fructose-6-phosphate transaminase (*glmS*, K00820), a key enzyme in bacterial cell wall and peptidoglycan precursor synthesis, was significantly elevated ( Fig. 3). Both findings indicate increased bacterial and fungal cell wall biosynthesis at the flowering growth stage.

Two catabolic gene transcripts were also responsive to growth stage. The peptidoglycan lytic transglycosylase D (K08307, *mltD* and *dniR*) and peptidoglycan lytic transglycosylase G (K07082) both showed increased transcript abundance at flowering, pointing to heightened peptidoglycan turnover, while transcripts associated with chitin and plant polymer degradation did not show a significant increase (Fig. S5B).

Only one transcript related to bacterial chemotaxis was significantly affected by growth stage: the chemotaxis protein *MotB* (K02557), which was highly elevated at flowering (*P* < 0.001) (Fig. S5C) . This suggests an increased capacity for directed bacterial movement in response to environmental conditions.

Three transcripts encoding enzymes involved in EPS production were upregulated at flowering. *ExoY* (K16566) exhibited the strongest response to growth stage (Fig. S5D) and is specific to EPS production in bacteria. The alginate O-acetyltransferase complex protein (K19296, *algF*), responsible for alginate synthesis, was also elevated at flowering. Additionally, serine O-acetyltransferase (K00640, *cysE*) was increased, indicating a broader activation of EPS-related pathways during flowering. This enzyme is responsible for cysteine synthesis and important for glycan and EPS production.

Transcripts associated with substrate transport were substantially affected by growth stage, with this category containing the highest number of affected transcripts. The xylose substrate-binding protein (K10543) and rhamnose S-B protein (K10559, *rhaS*) were both significantly elevated at flowering (p < 0.001). Alongside the trehalose/maltose transport system permease protein (K10237) and polyol transport system permease protein (K10229). The phospholipid/cholesterol/gamma-HCH transport system permease protein (K02066, *mlaE, linK*) was also upregulated, suggesting enhanced transport and metabolic flexibility during flowering (Fig. S5E).

The two-way ANOVA identified only two transcripts significantly affected by soil admixture. Glutamine-fructose-6-phosphate transaminase (*glmS* K00820), which was also influenced by growth stage, was stronglydecreased in the 24% admixture treatment, suggesting that this transcript was the main driver of differences between the 0% and 24% treatments. Furthermore, the bifunctional S-B protein of ribose and bacterial chemotaxis (K10439) was more abundant in the 0% admixture soil compared to the 24% admixture, indicating potential sensitivity of these pathways to soil erosion conditions. The selective decrease in the simulated admixture treatment suggests a specific response of these transcripts and a dilution effect for other transcripts.

### Tailored KEGG Orthologs (KO) analysis based on TPM-normalize reads

The normalized relative abundance (TPM) was highest for catabolic enzymes and ABC transporters. Across the groups, the TPM values were remarkably similar, indicating towards an overall low effect of growth stage and subsoil admixture on the total transcript abundance within functional groups of enzymes. Nonetheless, PERMANOVA was significant for both experimental factors, with a low effect strength. The PERMANOVA for the tailored KOs reads revealed that both growth stage and admixture explained around 5% of the differences (p < 0.05) with no effect of the interaction (Tables S2D). PCoA only showed weak separation with no clear clustering (Fig. S4D).

This was also reflected in a much lower number of TPM normalized reads showing a significant effect for either research factor. Only two transcripts were affected by growth stage and one by admixture. Specifically, K00698 associated with chitin synthase was elevated at the flowering growth stage. Likewise, K16566 (*ExoY)*, which is involved in bacterial EPS production, was more abundant at flowering. Admixture only affected *glmS* K00820 which is associated with PGN synthesis and was lower in the 24% admixture treatment (Appendix A).

### Taxonomic assignment of functional gene transcripts

**Figure 4:**
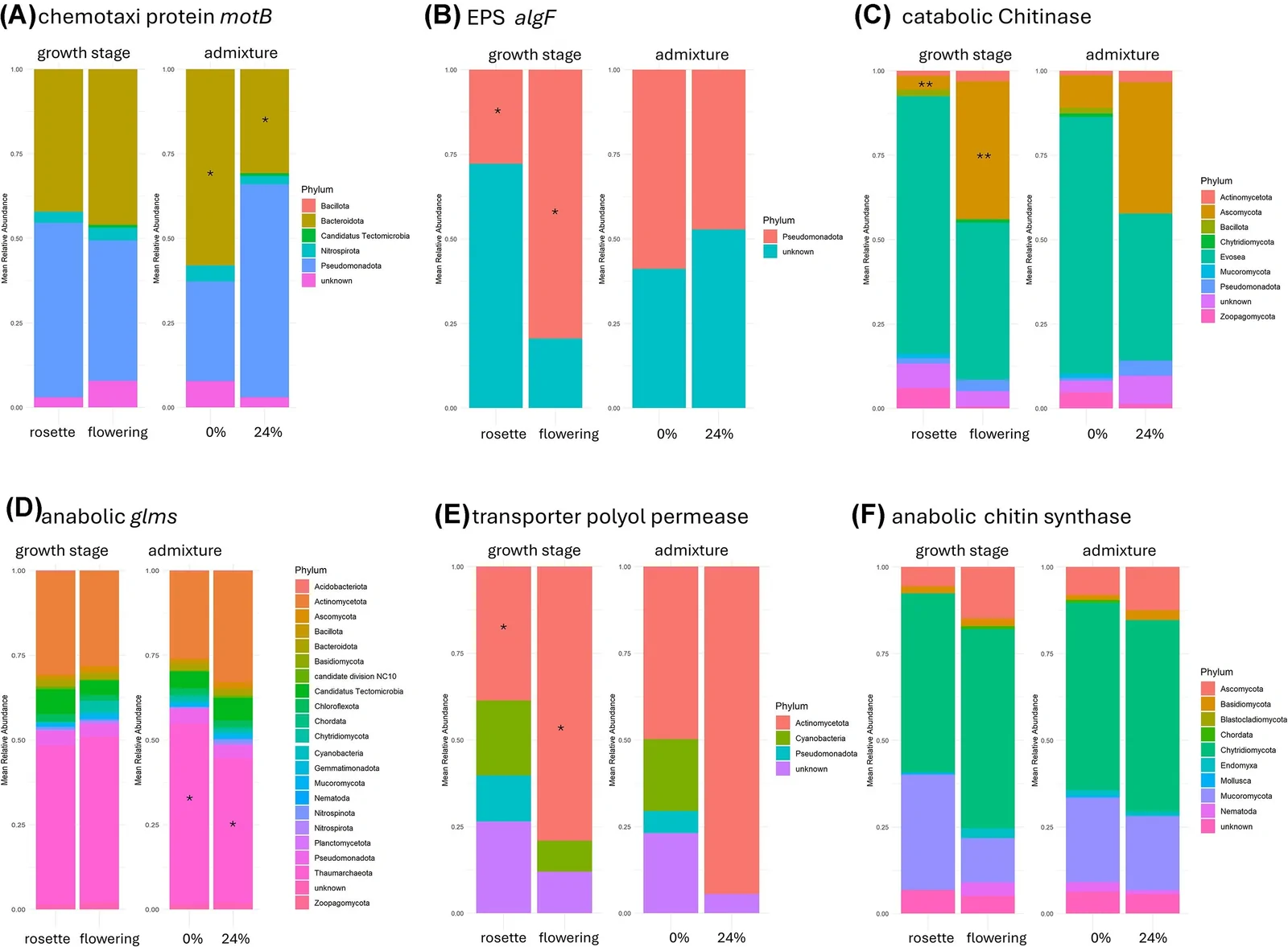
Taxonomic composition at the phylum level of KO-annotated transcripts corresponding to (A) chemotaxis protein *motB*, (B) *algF* related to EPS production, (C) Chitinase, (D) *glmS* precursor of PGN, (E) polyol permease, (F) chitin synthase. TPM abundances of the transcripts were log2(x +1) transformed before two-way ANOVA with FDR *P*-value correction with growth stage and admixture as factors. *, *P* < 0.05. **, *P* < 0.01. Values are untransformed mean TPM values translated to percentages.

We used the KO groups affected by growth stage or admixture treatment to determine whether there were changes in the taxonomic composition of the KO groups. The chemotaxis protein *motB* was mostly composed of transcripts assigned to *Bacteroidota* and *Pseudomonadota* comprising around 90% of all reads. An admixture effect was observed, with a significantly higher share of *Bacteroidota* in the 0% treatment compared to the 24% treatment (Fig. 4A). Transcripts of the *algF* gene were mostly assigned to *Pseudomonadota*, which was the only bacterial phyla assigned (Fig. 4B). It also showed a growth stage effect, higher abundance of *Pseudomonadota* were assigned in flowering with around 80% associated with this phylum. The remaining reads were unassigned. Chitinase-related transcripts were also investigated, and a strong increase in the abundance of *Ascomycota* was found at the flowering growth stage. This suggests fungal use of chitin as a substrate, either from other fungi or due to fungal senescence. Chitinase transcripts encompassed a wide variety of other phyla also present, such as *Evosea*, and some bacterial phyla. (Fig. 4C).

*glmS* transcripts were highly diverse and many different bacterial and archaeal phyla were found. *Thaumarchaeota* emerged as the most abundant taxonomic group among transcripts assigned to 23 bacterial, archaeal and fungal phyla associated with *glmS* (K00820), which encodes glutamine-fructose-6-phosphate transaminase, a key enzyme in PGN anabolism and other basic cellular functions (Fig. 4D). This is noteworthy given the otherwise low occurrence of archaea in the dataset. *Thaumarchaeota* comprised more than 50% of reads in the 0% admixture treatment, which was significantly higher than in the 24% admixture treatment. The polyol permease, which was also affected by growth stage, was overall dominated by the phylum *Actinomycetota*, which was significantly higher at flowering. Other phyla contributing were *Cyanobacteriota* and *Pseudomonota*. ( Fig. 4E)

Furthermore, Chitin synthase gene expression was investigated which had a surprisingly large share of transcripts assigned to *Chordata*, which can be attributed to small soil animals. *Ascomycota* was the most abundant fungal phylum at flowering for chitin synthase, while *Mucoromycota* was themost abundant for rosette growth stage suggesting a shift in fungi from the early to the later growth stage which was, however, not significant. (Fig. 4F)

Other significantly affected transcripts as described in Fig. 3 were also checked for taxonomic changes but no effect on the taxonomic composition was observed at the phylum level.

## Discussion

We were able to establish an mRNA-targeted approach based on KEGG Orthologs (KO) to detect quantitative functional changes in the metatranscriptome of the oilseed rape rhizosphere microbiome in response to growth stage and simulated soil erosion. We found pronounced effects of growth stage on the absolute functional transcript profiles, and a weaker effect on TPM-normalized transcripts for both KO-annotated and CAZy-annotated transcripts. Moreover, the absolute abundance of transcripts consistently increased at flowering, with a strong effect on ABC transporters, anabolic transcripts and transcript related to microbial cell wall degradition, but not plant polymer degradation. The experimentally simulated erosion caused only a limited effect on some transcript abundances of the functional categories that we analyzed, namely, *glmS* and the bifunctional *rbsB* were affected. Likewise, the interaction of both experimental factors had no effect, likely due to an overall small effect size of the two treatment factors on the functional metatranscriptome. TPM-normalized transcripts were also remarkably similar for the KO analysis. Some distinct taxonomic shifts were found for almost half of the selected functional transcripts, but in general, such compositional changes were minor. Furthermore, we used the CAZy database, which revealed a slightly higher number of families affected by growth stage and admixture. The results suggest that glucosidases and galactosidases were increased at flowering. However, due to the large number of enzymes summarized in one family, the conclusions that can be drawn from this part of the analysis are less specific to certain C pathways and exclude important enzymes responsible for EPS synthesis and substrate transport into prokaryotic cells.

### Increase of bacterial ABC transporter and plant polymer-degrading transcripts

We observed a marked increase in ABC transporter transcripts at flowering, with the strongest upregulation detected for the substrate-binding (S-B) protein of xylose. S-B proteins bind target substrates and deliver them to the permease, which functions as the transmembrane domain facilitating transport into the cytoplasm. The increased abundance of S-B protein transcripts may reflect the untethered nature of these proteins in Gram-negative bacteria, which allows for higher substrate uptake in nutrient-rich environments (Thomas and Tampé 2020). Specifically, the upregulation of S-B proteins for xylose and rhamnose, compounds ubiquitous in plant roots, suggests a selective microbial response to the rhizosphere environment during flowering (Koudounas 2021). *Brassica* crops are well documented to exude significant amounts of lipids (Chaparro et al. 2013, Vives-Peris et al. 2020, Han et al. 2024), which may explain the increased microbial capacity for phospholipid permease at the flowering growth stage. While phospholipid fatty acids are commonly used as indicators of microbial biomass in soils (Joergensen 2022), in the rhizosphere of oil crops the high concentrations of linoleic and oleic acids in plant exudates may be directly taken up by microbial cells. Trehalose, an important signaling molecule in plants that can be found in high concentrations in root clusters of some plants (Shane et al. 2016) appears to have a complex effect on the rhizosphere microbiome. Trehalose can decrease the abundance of beneficial soil microorganisms and inhibit biofilm formation in tea plants but is also an important molecule produced by bacteria for protection against viral infection (Vanaporn and Titball 2020, Zhu et al. 2026).

In summary, our results for bacterial transporters suggest a selective increase of S-B proteins and permeases at flowering with no apparent effect on the ATP-binding proteins. Interestingly, we did not find any effect of growth stage or admixture on plant polymer degradation, such as cellulases, pectinases or peroxidases degrading lignin, with the tailored KO analysis. However, a distinct effect was observed for the CAZy families related to such enzyme families. GH1, which was more abundant at the rosette growth stage and affected by admixture, was an abundant CAZy family in our metatranscriptome and includes β-galactosidase, β-glucosidase and xylan exo-β-1,4-xylosidase, which collectively mediate plant polymer degradation (Drula et al. 2022).

### Fungal and bacterial metabolic functions of cell wall anabolism and catabolism

We observed a pronounced effect on chitin synthase correlated with growth stage, highlighting the pivotal role of fungi in the rhizosphere. Our analysis did not find any transcripts belonging to the melanin or β-glucan synthesis pathways, although both are crucial components of the fungal cell wall and subsequent fungal-derived SOC (Ruiz-Herrera and Ortiz-Castellanos 2019, Camenzind et al. 2023). One explanation may be the relatively low abundance of fungi overall, together with the challenges encountered in soil metatranscriptomics, such as fragmentation and in our study specifically, the rRNA depletion of only bacteria and relatively low recovery of fungal markers (Appendix B).

Nonetheless, fungi are important for agricultural soils due to the fast growth rates of hyphae that act as pioneers in rhizosphere colonization, and rapid utilization of available substrates may lead to a rapid increase in chitin synthesis (Taylor and Sinsabaugh 2015, Begum et al. 2019). Fungi use compartmental senescence to discard hyphae that are no longer beneficial for growth. These senescent hyphae are often enriched in cell wall components that contribute to microbe-derived SOC through necromass formation (Camenzind et al. 2023). In our experiment, the abundance of *Mucoromycota* was higher than previously detected in *Brassica napus* by ITS metabarcoding using the same soil (Ruggaber et al. 2024).

The high taxonomic diversity of chitinase transcripts indicates that various groups of microorganisms act linked together and possibly form assemblages on dead hyphae. Chitin is enzymatically degraded by a large variety of microorganisms, as we also found, as the fungal cell wall polymer is an excellent source of C and N (Wieczorek et al. 2019). Endo- and exochitinases act in conjunction to cleave larger chitin polymers into dimers, which are transported through the cell membrane and used for energy generation in the Krebs cycle after dissociation from the chitin-binding protein (Nayak et al. 2021), highlighting the importance of the joint study of degrading enzymes with the corresponding substrate transporters.

The synthesis of peptidoglycan (PGN), the main cell wall component of bacteria, is a more complex biosynthetic pathway as compared with chitin, involving an array of enzymes, some of which are not specific for PGN synthesis (Garde et al. 2021). Bacteria are able to recycle intermediate compounds and allocate them for PGN synthesis (Garde et al. 2021). While *glmS* (K00820) is essential for PGN precursor synthesis, this enzyme is also involved in the synthesis of nucleotide sugars, amino sugars and some amino acids synthesis unrelated to PGN (Garde et al. 2021). It can be a rate-limiting step in PGN synthesis but is likely not a reliable indicator for PGN synthesis by itself. The *RodZ* enzyme, which is essential in maintaining the rod shape of bacterial cells, is likely a better single gene-based indicator for PGN synthesis. *RodZ* directs the *MreB* protein into the existing peptidoglycan structure; thus, it is directly involved in maintaining the bacterial cell wall (Morgenstein et al. 2015).

Likewise, the degradation of PGN involves many enzymes due to its chemical heterogeneity. We found a significant effect for chitinolytic transglycosylase G (K07082) and peptidoglycan lytic transglycosylase D (K08307), which cleave PGN (Humann and Lenz 2009). Thus, these enzymes are promising indicators for bacterial PGN degradation. Pseudopeptidoglycan, the archaeal equivalent of PGN, may be of importance in archaea-dominated soils but is difficult to investigate with our approach due to a lack of database entries for archaea (Subedi et al. 2021). In the CAZy reference database, the enzymes for chitin and PGN degradation are summarized in one family (GH23) based on their structural similarities (Drula et al. 2022), which was also found in our metatranscriptome. However, this grouping makes a clear distinction between fungal- and bacterial-derived cell wall activity challenging.

Further studies on bacterial biomass degradation and recycling in soils are required to identify the most suitable indicator genes or markers for the microbial turnover of this structural polymer. The addition of ^13^C-labelled substrate and DNA-based stable isotope probing (DNA-SIP) combined with our tailored KO approach might help to further separate which genes are involved in bacterial anabolism and catabolism of PGN, thereby fuelling the SOC pool (Buckeridge et al. 2020).

### EPS synthesis and bacterial chemotaxis

The transcript abundances of the exopolysaccharide production protein (*exoY*, K16566) and alginate-producing protein (*algF*, K19296) and cysE (K00640) which is involved in cysteine production were elevated at flowering. The synthesis of EPS such as colanic acid, which contains galactose and glucuronic acid, suggests potential pathways for the incorporation of these sugars into SOC, i.e. as precursors or directly as amino sugars (Oliva et al. 2024). While direct measurements of EPS in soils pose challenges due to the complex and transient nature of EPS, the assessment of chemical precursors or breakdown products may provide valuable indicators. Amino sugars, for example, are used as chemical indicators of microbial contributions to SOC, although their direct measurement may overestimate their importance (Nannipieri et al. 2025). Genes encoding enzymes responsible for EPS synthesis may be another proxy (Joergensen 2018). However, our approach only allowed an assessment of bacterial EPS transcripts, while fungi also use EPS for degradation of plant polymers (op de Beeck et al. 2021). Interestingly, we also found an increase in the abundance of PL7 in the CAZy-annotated transcripts, which is related to alginate-degrading enzymes.

A growth stage effect was observed for transcripts encoding the bacterial chemotaxis protein *motB* and an admixture effect for the bifunctional ribose S-B protein and chemotaxis protein *rbsB* (K10439), which has been known for long time to be of importance for chemotaxis activity (Björkman et al. 1994). Both effects may be explained by the bacterial response to changing substrate availability. Thus, chemotaxis appears to be a relevant indicator for bacterial motility in response to nutrient gradients.

### Prospective applications of metatranscriptomics to study microbial SOC turnover

The degradation of polymers such as pectin, lignin, chitin and hemicellulose has been frequently studied in soils, i.e. with soil enzyme assays (Sinsabaugh et al. 2008) or with nucleic acid analysis (Schellenberger et al. 2010, Wieczorek et al. 2019). However, metatranscriptomics allows for the simultaneous assessment of transcripts with various functional aspects that act jointly. The pronounced growth stage effect is likely explained by an overall more active rhizosphere microbiome of *Brassica napus* at flowering, as reflected by higher C_mic_ and RNA contents.

The limited representation of fungal genes in the reference databases highlights the need for further investigations into functional responses of fungi. The detection of functional groups of fungi is often focused on the assignment of ITS sequences and subsequent annotation of functional aspects of the encountered fungi (Põlme et al. 2020). While this provides some insights into the lifestyle of fungi, it does not resolve functional genes directly, as our approach did. Furthermore, fungal transmembrane transporters exhibit high diversity and are relatively poorly studied compared to bacterial transmembrane transporters, thus, they are another potential research target (Peng et al. 2018, Nogueira et al. 2020).

Our study suggests that quantitative metatranscriptomics has the potential to monitor the formation of amino sugars and other SOC formation markers in soil, shedding light on microbial processes that foster SOC storage and turnover (Angst et al. 2021). Future research can likely identify more genes that are of relevance for SOC formation in soils. The current databases have the best coverage for functional genes of bacteria, as opposed to the limited coverage of archaea and fungi in existing reference databases (Lind and Pollard 2021). This needs to be addressed in the future to further disentangle the in situ functions of these microbial groups in the rhizosphere (Zanne et al. 2020). A previously unfeasible metagenome-assembled genome soil database, comparable to the Human Genome Project, might pave the way towards an increased coverage of uncultured microorganisms in soil (Anthony et al. 2024). More specific databases, such as the LCdb for lignin degradation, are limited to specific substrates but could be included in a comprehensive database (J. Chen et al. 2024). This may also include the soil virome, in which there has been growing interest recently, as viruses and phages infect soil-borne microorganisms and thus, contribute to microbial death and necromass formation (Bi et al. 2021, Camenzind et al. 2023, Ma et al. 2024).

The weaker treatment effects observed for TPM-normalized abundances compared with transcripts g⁻¹ dw soil abundances may reflect the different information captured by these measures. TPM describes the relative composition of the transcript pool and removes differences in overall transcript abundance among samples, whereas transcripts g⁻¹ dw soil retain differences in the absolute magnitude of transcriptional activity. Likewise, the absolute approach allows the estimation of the magnitude of ecological processes, which has been applied before in other environments, such as marine environments and the rumen, but also in soils to quantify methanogen and methanotroph activity (Söllinger et al. 2018, Täumer et al. 2022, Gifford et al. 2011). We demonstrate the utility of absolute transcript quantification for understanding C turnover processes by the rhizosphere microbiome that eventually lead to SOC sequestration.

Furthermore, taxonomic annotation of functional reads introduces some degree of uncertainty, as assignments depend on the quality of the reference database and contig quality and may lead to ambiguous assignments. Thus, the reliability of taxonomic identification of functional genes can be considerably lower compared to metabarcoding of rRNA. Enrichment of reads of interest can also lead to higher recovery and longer contigs, thus improving taxonomic assignment of functional genes. Thus, the phylum level appeared to be most adequate due to increasing uncertainty at lower taxonomic levels.

Further refinement of the tailored KO approach may include targeted enrichment with a MetCap pipeline or custom oligonucleotides with hybridization probes that can enhance the proportion of sequences that are of relevance for the SMCP (Kushwaha et al. 2015, Manoharan et al. 2015, Kozjek et al. 2023). The approach could also be further tailored to fungi in environments where fungal activity is higher and address the specific challenges of eukaryotes. Our tailored approach and subsequent data pipeline appear most appropriate to study the transcriptome of bacteria- and archaea-dominated soils.

## Conclusions

The major objective of this study was to develop a methodological framework that links molecular-level information of the SMCP with plant-soil system-level research on SOC formation and persistence. Our study highlights functions of the rhizosphere microbiome that are poorly resolved by classical methods such as soil enzyme assays or metabarcoding. However, these functions are pivotal for advancing our understanding of the interplay between plant roots and the rhizosphere microbiome in SOC dynamics. We have shown that the simultaneous assessment of various metabolic functions is feasible and can be effectively tailored using KEGG orthologs (KOs). KOs proved superior for studying specific metabolic microbiome functions compared with the CAZy database, which comprises structurally similar enzyme families. Interestingly, we have found an increase in S-B proteins and permeases of bacterial ABC transporters as well as transcripts related to EPS production, at flowering, while no effect was found on transcripts encoding plant polymer-degrading enzymes. This suggests that mostly root exudates drive microbial growth in the rhizosphere. Simulated erosion only had a minor, modulatory effect in our setup, affecting two transcripts. CAZy results showed a similar pattern for increases in chitin and PGN-related anabolic transcripts and plant exudate utilization, but also in transcripts encoding plant polymer-degrading enzymes. Current databases and methods provide the best coverage of metabolic functions of bacteria; in our experiment, the evaluation of fungal functions was limited to the most prominent transcripts in our experiment. However, the role of fungi and archaea in many plant-soil systems may still be underestimated due to a lack of annotated genomes in public databases. Similarly, the taxonomic coverage of functional genes related to the SMCP is biased towards cultivable organisms. Quantitative metatranscriptomics, as applied here, can capture the host plant and soil effects (in this study, the simulated erosion state) on the dynamics of microbiome functions involved in SOC formation. This approach provides a basis to identify further functional gene markers that can characterize anabolic and catabolic processes of the SMCP in future research.

## Supplementary Material

fiag097_Supplemental_Files

## Data Availability

Raw reads of the samples are available on NCBI’s SRA under BioProject id PRJNA1140101. Scripts for data aggregation are available on https://github.com/j-ruggaber-zalf/Metatranscriptome_aggregation_scripts.

## References

[bib1] Angst G, Mueller KE, Nierop KGJ. et al. Plant- or microbial-derived? A review on the molecular composition of stabilized soil organic matter. Soil Biol Biochem. 2021;156:108189. 10.1016/j.soilbio.2021.108189.

[bib2] Anthony WE, Allison SD, Broderick CM. et al. from soil to sequence: filling the critical gap in genome-resolved metagenomics is essential to the future of soil microbial ecology. Environmental Microbiome. 2024;19:56. 10.1186/s40793-024-00599-w. PMC11295382 39095861

[bib3] Apprill A, McNally S, Parsons R. et al. Minor revision to V4 region SSU rRNA 806R gene primer greatly increases detection of SAR11 bacterioplankton. Aquat Microb Ecol. 2015;75:129–37. 10.3354/ame01753.

[bib4] Artzi L, Bayer EA, Moraïs S. Cellulosomes: bacterial nanomachines for dismantling plant polysaccharides. Nat Rev Microbiol. 2017;15:83–95. 10.1038/nrmicro.2016.164. 27941816

[bib5] Begill N, Don A, Poeplau C. No detectable upper limit of mineral-associated organic carbon in temperate agricultural soils. Global Change Biol. 2023;29:4662–9. 10.1111/gcb.16804. 37271832

[bib6] Begum N, Qin C, Ahanger MA. et al. Role of arbuscular mycorrhizal fungi in plant growth regulation: implications in abiotic stress tolerance. Front Plant Sci. 2019;10:1068. 10.3389/fpls.2019.01068. PMC6761482 31608075

[bib7] Bi L, Yu D-T, Du S. et al. Diversity and potential biogeochemical impacts of viruses in bulk and rhizosphere soils. Environ Microbiol. 2021;23:588–99. 10.1111/1462-2920.15010. 32249528

[bib8] Björkman AJ, Binnie RA, Zhang H. et al. Probing protein-protein interactions: the ribose-binding protein in bacterial transport and chemotaxis. J Biol Chem. 1994;269:30206–11. 10.1016/S0021-9258(18)43798-2. 7982928

[bib9] Blin K, Shaw S, Medema MH. et al. the antiSMASH database version 4: additional genomes and BGCs, new sequence-based searches and more. Nucleic Acids Res. 2024;52:D586–9. 10.1093/nar/gkad984. PMC10767862 37904617

[bib10] Brookes PC, Landman A, Pruden G. et al. Chloroform fumigation and the release of soil nitrogen: a rapid direct extraction method to measure microbial biomass nitrogen in soil. Soil Biol Biochem. 1985;17:837–42. 10.1016/0038-0717(85)90144-0.

[bib11] Buchfink B, Reuter K, Drost H-G. Sensitive protein alignments at tree-of-life scale using DIAMOND. Nat Methods. 2021;18:366–8. 10.1038/s41592-021-01101-x. PMC8026399 33828273

[bib12] Buckeridge KM, Mason KE, McNamara NP. et al. Environmental and microbial controls on microbial necromass recycling, an important precursor for soil carbon stabilization. Communications Earth & Environment. 2020;1:31. 10.1038/s43247-020-00031-4.

[bib13] Bushnell B. BBMap: A Fast, Accurate, Splice-Aware Aligner. 9th Annual Genomics of Energy & Environment Meeting, Walnut Creek, CA. 2014.

[bib14] Camenzind T, Mason-Jones K, Mansour I. et al. Formation of necromass-derived soil organic carbon determined by microbial death pathways. Nat Geosci. 2023;16:115–22. 10.1038/s41561-022-01100-3.

[bib15] Canarini A, Kaiser C, Merchant A. et al. Root exudation of primary metabolites: mechanisms and their roles in plant responses to environmental stimuli. Front Plant Sci. 2019;10:157. 10.3389/fpls.2019.00157. PMC6407669 30881364

[bib16] Cantarel BL, Coutinho PM, Rancurel C. et al. the Carbohydrate-Active EnZymes database (CAZy): an expert resource for glycogenomics. Nucleic Acids Res. 2009;37(D233–8. 10.1093/nar/gkn663. PMC2686590 18838391

[bib17] Carvalhais LC, Dennis PG, Tyson GW. et al. Application of metatranscriptomics to soil environments. J Microbiol Methods. 2012;91:246–51. 10.1016/j.mimet.2012.08.011. 22963791

[bib18] Chaparro JM, Badri DV, Bakker MG. et al. Root exudation of phytochemicals in Arabidopsis follows specific patterns that are developmentally programmed and correlate with soil microbial functions. PLoS One. 2013;8:e55731. 10.1371/journal.pone.0055731. PMC3562227 23383346

[bib19] Chen S. Ultrafast one-pass FASTQ data preprocessing, quality control, and deduplication using fastp. iMeta. 2023;2:e107. 10.1002/imt2.107. PMC10989850 38868435

[bib20] Chen W, Cao P, Liu Y. et al. Structural basis for directional chitin biosynthesis. Nature. 2022;610:402–8. 10.1038/s41586-022-05244-5. PMC9556331 36131020

[bib21] Chen J, Lin L, Tu Q. et al. Metagenomic-based discovery and comparison of the lignin-degrading potential of microbiomes in aquatic and terrestrial ecosystems via the LCdb database. Mol Ecol Resour. 2024;24:e13950. 10.1111/1755-0998.13950. 38567644

[bib22] Colin R, Ni B, Sourjik V. Multiple functions of flagellar motility and chemotaxis in bacterial physiology. FEMS Microbiol Rev. 2021;45:fuab038. 10.1093/femsre/fuab038. PMC8632791 34227665

[bib23] Costa OYA, Raaijmakers JM, Kuramae EE. Microbial extracellular polymeric substances: ecological function and impact on soil aggregation. Front Microbiol. 2018;9:1636. 10.3389/fmicb.2018.01636. PMC6064872 30083145

[bib24] Deng Z-L, Münch PC, Mreches R. et al. Rapid and accurate identification of ribosomal RNA sequences via deep learning. Nucleic Acids Res. 2022;50:e60. 10.1093/nar/gkac112. PMC9177968 35188571

[bib25] Drula E, Garron M-L, Dogan S. et al. the carbohydrate-active enzyme database: functions and literature. Nucleic Acids Res. 2022;50:D571–7. 10.1093/nar/gkab1045. PMC8728194 34850161

[bib26] Flemming H-C, Wingender J. the biofilm matrix. Nat Rev Microbiol. 2010;8:623–33. 10.1038/nrmicro2415. 20676145

[bib27] Garde S, Chodisetti PK, Reddy M. Peptidoglycan: structure, synthesis, and regulation. EcoSal Plus. 2021;9:ESP–0010-2020. 10.1128/ecosalplus.ESP-0010-2020. PMC11168573 33470191

[bib28] Georgiou K, Jackson RB, Vindušková O. et al. Global stocks and capacity of mineral-associated soil organic carbon. Nat Commun. 2022;13:3797. 10.1038/s41467-022-31540-9. PMC9249731 35778395

[bib29] Gifford SM, Sharma S, Rinta-Kanto JM. et al. Quantitative analysis of a deeply sequenced marine microbial metatranscriptome. ISME J. 2011;5:461–72. 10.1038/ismej.2010.141 PMC3105723 20844569

[bib30] Grabherr MG, Haas BJ, Yassour M. et al. Full-length transcriptome assembly from RNA-seq data without a reference genome. Nat Biotechnol. 2011;29:644–52. 10.1038/nbt.1883. PMC3571712 21572440

[bib31] Gurevich A, Saveliev V, Vyahhi N. et al. QUAST: quality assessment tool for genome assemblies. Bioinformatics. 2013;29:1072–5. 10.1093/bioinformatics/btt086. PMC3624806 23422339

[bib32] Han Y, Teng Y, Wang X. et al. Biodegradable PBAT microplastics adversely affect pakchoi (Brassica chinensis L.) growth and the rhizosphere ecology: focusing on rhizosphere microbial community composition, element metabolic potential, and root exudates. Sci Total Environ. 2024;912:169048. 10.1016/j.scitotenv.2023.169048. 38061654

[bib33] Harden JW, Sharpe JM, Parton WJ. et al. Dynamic replacement and loss of soil carbon on eroding cropland. Global Biogeochem Cycles. 1999;13:885–901. 10.1029/1999GB900061.

[bib34] Humann J, Lenz LL. Bacterial peptidoglycan degrading enzymes and their impact on host muropeptide detection. Journal of Innate Immunity. 2009;1:88–97. 10.1159/000181181. PMC2659621 19319201

[bib35] Huson DH, Beier S, Flade I. et al. MEGAN community edition-interactive exploration and analysis of large-scale microbiome sequencing data. PLoS Comput Biol. 2016;12:e1004957. 10.1371/journal.pcbi.1004957 PMC4915700 27327495

[bib36] Hyatt D, Chen GL, LoCascio PF. et al. Prodigal: prokaryotic gene recognition and translation initiation site identification. BMC Bioinf. 2010;11:119. 10.1186/1471-2105-11-119. PMC2848648 20211023

[bib37] Joergensen RG. Amino sugars as specific indices for fungal and bacterial residues in soil. Biol Fertil Soils. 2018;54:559–68. 10.1007/s00374-018-1288-3.

[bib38] Joergensen RG. Phospholipid fatty acids in soil—Drawbacks and future prospects. Biol Fertil Soils. 2022;58:1–6. 10.1007/s00374-021-01613-w.

[bib39] Kandeler E. Physiological and biochemical methods for studying soil biota and their functions. In Paul F. E., Paul E. A., Myrold D. (Eds.), Soil microbiology, ecology and biochemistry. Elsevier. 2024, 193–227.

[bib40] Kanehisa M. the KEGG database In Bock G., Goode J. A. (Eds.), ‘in silico’ simulation of biological processes. Wiley. 2002;247, 91–103. 10.1002/0470857897.ch8.

[bib41] Karaoz U, Emerson JB, Brodie EL. Molecular and associated approaches for studying soil biota and their functioning. In Paul F. E., Paul E. A., Myrold D. (Eds.), Soil microbiology, ecology and biochemistry, 5th ed., Elsevier. 2024, 161–92.

[bib42] Kaštovská E, Choma M, Angst G. et al. Root but not shoot litter fostered the formation of mineral-associated organic matter in eroded arable soils. Soil Tillage Res. 2024;235:105871. 10.1016/j.still.2023.105871.

[bib43] Koudounas K. Players in pectin production: rhamnose transporters affect the length of rhamnogalacturonan-I. Plant Physiol. 2021;185:759–60. 10.1093/plphys/kiaa104. PMC8133624 33793931

[bib44] Kozjek K, Manoharan L, Urich T. et al. Microbial gene activity in straw residue amendments reveals carbon sequestration mechanisms in agricultural soils. Soil Biol Biochem. 2023;179:108994. 10.1016/j.soilbio.2023.108994.

[bib45] Kushwaha SK, Manoharan L, Meerupati T. et al. MetCap: a bioinformatics probe design pipeline for large-scale targeted metagenomics. BMC Bioinf. 2015;16:65. 10.1186/s12859-015-0501-8. PMC4355349 25880302

[bib46] Taylor DL, Sinsabaugh RL. The soil fungi. In Paul F. E., Paul E. A., Myrold D. (Eds.), Soil microbiology, ecology and biochemistry, 5th ed., Elsevier. 2015, 77–109.

[bib47] Li M, Jiang C, Wang Q. et al. Evolution and functional insights of different ancestral orthologous clades of chitin synthase genes in the fungal tree of life. Front Plant Sci. 2016;7:37. 10.3389/fpls.2016.00037. PMC4734345 26870058

[bib48] Liang C. Soil microbial carbon pump: mechanism and appraisal. Soil Ecology Letters. 2020;2:241–54. 10.1007/s42832-020-0052-4.

[bib49] Liang C, Schimel JP, Jastrow JD. the importance of anabolism in microbial control over soil carbon storage. Nat Microbiol. 2017;2:17105. 10.1038/nmicrobiol.2017.105. 28741607

[bib50] Lind AL, Pollard KS. Accurate and sensitive detection of microbial eukaryotes from whole metagenome shotgun sequencing. Microbiome. 2021;9:58. 10.1186/s40168-021-01015-y. PMC7931531 33658077

[bib51] Ling N, Wang T, Kuzyakov Y. Rhizosphere bacteriome structure and functions. Nat Commun. 2022;13:836. 10.1038/s41467-022-28448-9. PMC8837802 35149704

[bib52] Ma B, Wang Y, Zhao K. et al. Biogeographic patterns and drivers of soil viromes. Nat Ecol Evol. 2024;8:717–28. 10.1038/s41559-024-02347-2. 38383853

[bib53] Manoharan L, Kushwaha SK, Hedlund K. et al. Captured metagenomics: large-scale targeting of genes based on “sequence capture” reveals functional diversity in soils. DNA Res. 2015;22:451–60. 10.1093/dnares/dsv026. PMC4675713 26490729

[bib54] McMurdie PJ, Holmes S. phyloseq: an R package for reproducible interactive analysis and graphics of microbiome census data. PLoS One. 2013;8:e61217. 10.1371/journal.pone.0061217. PMC3632530 23630581

[bib55] Morgenstein RM, Bratton BP, Nguyen JP. et al. RodZ links MreB to cell wall synthesis to mediate MreB rotation and robust morphogenesis. Proc Nat Acad Sci USA. 2015;112:12510–5. 10.1073/pnas.1509610112. PMC4603514 26396257

[bib56] Nannipieri P, Angst G, Mueller C. et al. the role of death and lysis of microbial and plant cells in the formation of soil organic matter. Soil Biol Biochem. 2025;204:109750. 10.1016/j.soilbio.2025.109750.

[bib57] Nayak SK., Nayak S., Mohanty S. et al. Mohapatra S., Samantaray D. (Eds.), Environmental and agricultural microbiology. Wiley. 2021, 313–40.

[bib58] Nguyen C. Rhizodeposition of organic C by plant: mechanisms and controls. In Lichtfouse E., Navarrete M., Debaeke P., Souchere V., Alberola C. (Eds.), Sustainable agriculture. Springer. 2009, 97–123. 10.1007/978-90-481-2666-8_7.

[bib59] Nogueira KMV, Mendes V, Carraro CB. et al. Sugar transporters from industrial fungi: key to improving second-generation ethanol production. Renewable Sustainable Energy Rev. 2020;131:109991. 10.1016/j.rser.2020.109991.

[bib60] Oliva RL, Khadka UB, Camenzind T. et al. Constituents of extracellular polymeric substances (EPS) produced by a range of soil bacteria and fungi. BMC Microbiol. 2025;25:298. 10.1186/s12866-025-04034-z. PMC12079940 40375143

[bib61] Oliva RL, Vogt C, Bublitz TA. et al. Galactosamine and mannosamine are integral parts of bacterial and fungal extracellular polymeric substances. ISME Communications. 2024;4:ycae038. 10.1093/ismeco/ycae038. PMC11014887 38616925

[bib62] op de Beeck M, Persson P, Tunlid A. Fungal extracellular polymeric substance matrices: highly specialized microenvironments that allow fungi to control soil organic matter decomposition reactions. Soil Biol Biochem. 2021;159:108304. 10.1016/j.soilbio.2021.108304.

[bib63] Parada AE, Needham DM, Fuhrman JA. Every base matters: assessing small subunit rRNA primers for marine microbiomes with mock communities, time series and global field samples. Environ Microbiol. 2016;18:1403–14. 10.1111/1462-2920.13023. 26271760

[bib64] Pausch J, Kuzyakov Y. Carbon input by roots into the soil: quantification of rhizodeposition from root to ecosystem scale. Global Change Biol. 2018;24:1–12. 10.1111/gcb.13850. 28752603

[bib65] Peng M, Aguilar-Pontes MV, de Vries RP. et al. in silico analysis of putative sugar transporter genes in Aspergillus niger using phylogeny and comparative transcriptomics. Front Microbiol. 2018;9:1045. 10.3389/fmicb.2018.01045. PMC5968117 29867914

[bib66] Põlme S, Abarenkov K, Nilsson RH. et al. FungalTraits: a user-friendly traits database of fungi and fungus-like stramenopiles. Fungal Diversity. 2020;105:1–16. 10.1007/s13225-020-00466-2.

[bib67] Rathore AS, Gupta RD. Chitinases from bacteria to humans: properties, applications, and future perspectives. Enzyme Research. 2015;2015:791907. 10.1155/2015/791907. PMC4668315 26664744

[bib68] R Core Team R: a language and environment for statistical computing. R version 4.2.1 (2022–06–23 ucrt). Vienna: R Foundation for Statistical Computing, 2022. https://www.R-project.org.

[bib69] Remus R, Kaiser M, Kleber M. et al. Demonstration of the rapid incorporation of carbon into protective, mineral-associated organic carbon fractions in an eroded soil from the CarboZALF experimental site. Plant Soil. 2018;430:329–48. 10.1007/s11104-018-3724-4.

[bib70] Ruggaber J, Pehlivan A, Remus R. et al. Simulated soil erosion predominantly affects fungal abundance in the rapeseed rhizosphere. Rhizosphere, 2024;30;100912. 10.1016/j.rhisph.2024.100912.

[bib71] Ruiz-Herrera J, Ortiz-Castellanos L. Cell wall glucans of fungi: a review. The Cell Surface. 2019;5:100022. 10.1016/j.tcsw.2019.100022. PMC7389562 32743138

[bib72] Rumpel C, Amiraslani F, Chenu C. et al. the 4 per 1000 initiative: opportunities, limitations and challenges for implementing soil organic carbon sequestration as a sustainable development strategy. Ambio. 2020;49:350–60. 10.1007/s13280-019-01165-2. PMC6889108 30905053

[bib73] Scharf BE, Hynes MF, Alexandre GM. Chemotaxis signaling systems in model beneficial plant-bacteria associations. Plant Mol Biol. 2016;90:549–59. 10.1007/s11103-016-0432-4. 26797793

[bib74] Schellenberger S, Kolb S, Drake HL. Metabolic responses of novel cellulolytic and saccharolytic agricultural soil bacteria to oxygen. Environ Microbiol. 2010;12:845–61. 10.1111/j.1462-2920.2009.02128.x. 20050868

[bib75] Shane MW, Feil R, Lunn JE. et al. Light-dependent activation of phospho enol pyruvate carboxylase by reversible phosphorylation in cluster roots of white lupin plants: diurnal control in response to photosynthate supply. Ann Bot. 2016;118:637–43. 10.1093/aob/mcw040 PMC5055616 27063365

[bib76] Sinsabaugh RL, Lauber CL, Weintraub MN. et al. Stoichiometry of soil enzyme activity at global scale. Ecol Lett. 2008;11:1252–64. 10.1111/j.1461-0248.2008.01245.x. 18823393

[bib77] Sofo A, Zanella A, Ponge J-F. Soil quality and fertility in sustainable agriculture, with a contribution to the biological classification of agricultural soils. Soil Use and Management. 2022;38:1085–112. 10.1111/sum.12702.

[bib78] Söllinger A, Tveit AT, Poulsen M. et al. Holistic assessment of rumen microbiome dynamics through quantitative metatranscriptomics reveals multifunctional redundancy during key steps of anaerobic feed degradation. mSystems. 2018;3:e00038–18. 10.1128/mSystems.00038-18. PMC6081794 30116788

[bib79] Sommer M, Augustin J, Kleber M. Feedbacks of soil erosion on SOC patterns and carbon dynamics in agricultural landscapes—the CarboZALF experiment. Soil Tillage Res. 2016;156:182–4. 10.1016/j.still.2015.09.015.

[bib80] Stewart CE, Paustian K, Conant RT. et al. Soil carbon saturation: concept, evidence and evaluation. Biogeochemistry. 2007;86:19–31. 10.1007/s10533-007-9140-0.

[bib81] Subedi BP, Martin WF, Carbone V. et al. Archaeal pseudomurein and bacterial murein cell wall biosynthesis share a common evolutionary ancestry. FEMS Microbes. 2021;2:xtab012. 10.1093/femsmc/xtab012. PMC10117817 37334239

[bib82] Tang L, Zhan M, Shang C. et al. Dynamics of root exuded carbon and its relationships with root traits of rapeseed and wheat. Plant, Soil and Environment. 2021;67:317–23. 10.17221/561/2020-PSE.

[bib83] Täumer J, Marhan S, Groß V. et al. Linking transcriptional dynamics of CH4-cycling grassland soil microbiomes to seasonal gas fluxes. ISME J. 2022;16:1788–97. 10.1038/s41396-022-01229-4. PMC9213473 35388141

[bib84] Tegenfeldt F, Kuznetsov D, Manni M. et al. OrthoDB and BUSCO update: annotation of orthologs with wider sampling of genomes. Nucleic Acids Res. 2025;53:D516–22. 10.1093/nar/gkae987. PMC11701741 39535043

[bib85] Thomas C, Tampé R. Structural and mechanistic principles of ABC transporters. Annu Rev Biochem. 2020;89:605–36. 10.1146/annurev-biochem-011520-105201. 32569521

[bib86] Tian H, Song H, Wu X. et al. Responses of cell wall components to low nitrogen in rapeseed roots. Agronomy. 2022;12:1044. 10.3390/agronomy12051044.

[bib87] Vanaporn M, Titball RW. Trehalose and bacterial virulence. Virulence. 2020;11:1192–202. 10.1080/21505594.2020.1809326 PMC7549927 32862781

[bib88] Vance ED, Brookes PC, Jenkinson DS. an extraction method for measuring soil microbial biomass C. Soil Biol Biochem. 1987;19:703–7. 10.1016/0038-0717(87)90052-6.

[bib89] van der Meij WM, Temme AJAM, Wallinga J. et al. Topography reconstruction of eroding landscapes: a case study from a hummocky ground moraine (CarboZALF-D). Geomorphology. 2017;295:758–72. 10.1016/j.geomorph.2017.08.015.

[bib90] Vives-Peris V, de Ollas C, Gómez-Cadenas A. et al. Root exudates: from plant to rhizosphere and beyond. Plant Cell Rep. 2020;39:3–17. 10.1007/s00299-019-02447-5. 31346716

[bib91] Vollmer W, Joris B, Charlier P. et al. Bacterial peptidoglycan (murein) hydrolases. FEMS Microbiol Rev. 2008;32:259–86. 10.1111/j.1574-6976.2007.00099.x. 18266855

[bib92] Wang B, An S, Liang C. et al. Microbial necromass as the source of soil organic carbon in global ecosystems. Soil Biol Biochem. 2021;162:108422. 10.1016/j.soilbio.2021.108422.

[bib93] Wang Q, Ding J, Zhang Z. et al. Rhizosphere as a hotspot for microbial necromass deposition into the soil carbon pool. J Ecol. 2025;113:168–79. 10.1111/1365-2745.14448.

[bib94] Wang X, Xu X, Qiu S. et al. Deep tillage enhanced soil organic carbon sequestration in China: a meta-analysis. J Cleaner Prod. 2023;399:136686. 10.1016/j.jclepro.2023.136686.

[bib95] Wenzel WW, Duboc O, Golestanifard A. et al. Soil and land use factors control organic carbon status and accumulation in agricultural soils of Lower Austria. Geoderma. 2022;409:115595. 10.1016/j.geoderma.2021.115595.

[bib96] Wieczorek AS, Schmidt O, Chatzinotas A. et al. Ecological functions of agricultural soil bacteria and microeukaryotes in chitin degradation: a case study. Front Microbiol. 2019;10:1293. 10.3389/fmicb.2019.01293. PMC6596343 31281293

[bib97] Wilkens S. Structure and mechanism of ABC transporters. F1000Prime Reports. 2015;7:14. 10.12703/P7-14. PMC4338842 25750732

[bib98] Wickham H. Getting Started with ggplot2. In ggplot2: Elegant graphics for data analysis. ggplot2. 2016;11–31.

[bib99] Zanne AE, Abarenkov K, Afkhami ME. et al. Fungal functional ecology: bringing a trait-based approach to plant-associated fungi. Biol Rev. 2020;95:409–33. 10.1111/brv.12570. 31763752

[bib100] Zeng Y, Charkowski AO. the role of ATP-binding cassette transporters in bacterial phytopathogenesis. Phytopathology. 2021;111:600–10. 10.1094/PHYTO-06-20-0212-RVW. 33225831

[bib101] Zhalnina K, Louie KB, Hao Z. et al. Dynamic root exudate chemistry and microbial substrate preferences drive patterns in rhizosphere microbial community assembly. Nat Microbiol. 2018;3:470–80. 10.1038/s41564-018-0129-3. 29556109

[bib102] Zheng J, Ge Q, Yan Y. et al. dbCAN3: automated carbohydrate-active enzyme and substrate annotation. Nucleic Acids Res. 2023;51:W115–21. 10.1093/nar/gkad328. PMC10320055 37125649

[bib103] Zhu Q, Chen B, Hu W. et al. Trehalose-mediated reshaping of the rhizosphere microbiome drives tea root rot progression. Front Microbiol. 2026;17:1787317. 10.3389/fmicb.2026.1787317 PMC12961617 41800398

