# Supplementary material for: Functional metatranscriptomics of the rhizosphere: towards an understanding of metabolic processes of the microbial carbon pump
Source: FEMS Microbiol Ecol. 2026 Sep 2;102(9):fiag097. doi: 10.1093/femsec/fiag097 (PMC13556623; doi:10.1093/femsec/fiag097)
Supplement: Supplementary file 1 [file fiag097_supplemental_files.zip › 20260819_Supp_tables_figures_Ruggaber.docx]

Supplementary Information:

**Functional metatranscriptomics of the rhizosphere: towards an understanding of metabolic processes of the microbial carbon pump**

Julian Ruggaber^a,b^, Sonja Wende^a^; Sizhong Yang^a^; Marco Keiluweit^c^, Steffen Kolb^a,b^*

*^a^* *Leibnizzentrum für Agrarlandschaftsforschung (ZALF) e.V., Müncheberg, Germany*

^b^ *Thaer Institute, Faculty of Life Sciences, Humboldt University of Berlin, Berlin, Germany*

^c^ *University of Lausanne, Faculty of Geosciences and the Environment, Lausanne, Switzerland*

**Corresponding author:* Steffen Kolb [kolb@zalf.de](mailto:kolb@zalf.de)
**Supplementary Method S1** Calculation of absolute transcript abundance of KO and CAZy annotated transcripts

The following calculation was used to calculate the absolute transcript abundance of transcripts * g^-1^ dw soil in a given sample. The approach was designed by (Söllinger et al., 2018) for transcript quantification per g^-1^ rumen fluid in cows, and applied to soil by (Täumer et al., 2021).

$$transcript A=total RNA \times\frac{mRNAr}{mRNAr+rRNAr}\times\frac{transcript Ar}{mRNAr}\times\frac{Na}{M \left( Nt \right)\times transcript A length}$$

In which:

transcript 𝐴 = absolute abundance of a given transcript (copies * g^-1^ dw soil)

total RNA = total RNA extracted (ng RNA * g^-1^ dw soil)

mRNAr = number of mRNA reads in a sample

rRNAr = number of rRNA reads used filtered before functional annotation

transcript *A_r_ =* number of reads assigned to a certain transcript

Na = Avogadro constant (6.02214076 × 10^23^ mol^-1^)

*M*(Nt) = average molecular weight of a single-stranded DNA (ssDNA) nucleotide (330 × 10^6^ µg·mol^−1^)

transcript A length = An average values of 1,000 nucleotides for mRNA

Söllinger, A., Tveit, A. T., Poulsen, M., Noel, S. J., Bengtsson, M., Bernhardt, J., … (2018). Holistic assessment of rumen microbiome dynamics through quantitative metatranscriptomics reveals multifunctional redundancy during key steps of anaerobic feed degradation. mSystems, 3(4), e00038–18. https://doi.org/10.1128/mSystems.00038-18

Täumer, J., Marhan, S., Groß, V., Jensen, C., Kuss, A. W., Kolb, S., & Urich, T. (2022). Linking transcriptional dynamics of CH4-cycling grassland soil microbiomes to seasonal gas fluxes. The ISME Journal, 16(7), 1788-1797. <https://doi.org/10.1038/s41396-022-01229-4>

Table S1. Soil nutrients as measured in the Ap and Bt horizon before the experiment in the ZALF central lab. DL = double lactate method.

| soil horizon | **% TC** | **% TOC** | **% N_total_** | **% S_total_** | **P_DL_ mg/100g** | **K_DL_ mg/100g** | **Mg_CaCl2_ mg/100g** | **pH  (CaCl_2_)** |
| --- | --- | --- | --- | --- | --- | --- | --- | --- |
| Ap | 0.97 | 0.86 | 0.10 | 0.01 | 7.66 | 12.82 | 5.03 | 7.39 |
| Bt | 0.17 | 0.16 | 0.03 | 0.01 | 0.63 | 5.85 | 5.95 | 6.51 |

**Table S2.** PERMANOVA results for functional composition (significance codes: 0 ‘***’ 0.001 ‘**’ 0.01 ‘*’ 0.05 ‘.’ 0.1 )

Table S2A PERMANOVA based on CAZy-annotated transcripts on level 2 calculated as transcripts * g ^-1^ dw soil

|  | **Df** | **SumOfSqs** | **R2** | **F** | **Pr(>F)** |
| --- | --- | --- | --- | --- | --- |
| **GrowthStage** | 1 | 0.14023 | 0.05831 | 2.0455 | 0.0125 * |
| **admixture** | 1 | 0.08295 | 0.03449 | 1.2101 | 0.2062 |
| **GrowthStage:admixture** | 1 | 0.05668 | 0.02357 | 0.8268 | 0.6689 |
| **Residual** | 31 | 2.12517 | 0.88363 |  |  |
| **Total** | 34 | 2.40503 | 1.00000 |  |  |

**Table S2B** PERMANOVA based on all KO-annotated transcripts in the metatranscriptome calculated as transcripts * g ^-1^ dw soil

|  | **Df** | **SumOfSqs** | **R2** | **F** | **Pr(>F)** |
| --- | --- | --- | --- | --- | --- |
| **GrowthStage** | 1 | 0.33693 | 0.13025 | 5.0494 | 0.001 *** |
| **admixture** | 1 | 0.12221 | 0.04724 | 1.8316 | 0.0076 ** |
| **GrowthStage:admixture** | 1 | 0.05922 | 0.02289 | 0.8875 | 0.6577 |
| **Residual** | 31 | 2.06851 | 0.79962 |  |  |
| **Total** | 34 | 2.58687 | 1.00000 |  |  |

|  | **Df** | **SumOfSqs** | **R2** | **F** | **Pr(>F)** |
| --- | --- | --- | --- | --- | --- |
| **GrowthStage** | 1 | 0.3374 | 0.07460 | 2.6654 | 0.0026 ** |
| **admixture** | 1 | 0.1189 | 0.02629 | 0.9394 | 0.4933 |
| **GrowthStage:admixture** | 1 | 0.1423 | 0.03147 | 1.1242 | 0.3060 |
| **Residual** | 31 | 3.9247 | 0.86764 |  |  |
| **Total** | 34 | 4.5234 | 1.00000 |  |  |

**Table S2C** PERMANOVA results based on tailored KO-annotated transcripts calculated as transcripts * g ^-1^ dw soil

|  | **Df** | **SumOfSqs** | **R2** | **F** | **Pr(>F)** |
| --- | --- | --- | --- | --- | --- |
| **GrowthStage** | 1 | 0.07187 | 0.04983 | 1.7616 | 0.0042 ** |
| **admixture** | 1 | 0.06679 | 0.04630 | 1.6370 | 0.0106 * |
| **GrowthStage:admixture** | 1 | 0.03906 | 0.02708 | 0.9573 | 0.5363 |
| **Residual** | 31 | 1.26479 | 0.87680 |  |  |
| **Total** | 34 | 1.44251 | 1.00000 |  |  |

**Table S2D** PERMANOVA results based on tailored KO (TPM normalized)

**Figure S1** Bioinformatic workflow highlighting all major steps performed. RNA was rRNA depleted before sequencing on 2x 150 bp paired end Illumnia Nova Seq. Subsequently raw reads were quality controlled and rRNA was removed. Unassembled reads were used for taxonomic assignment. Trinity was used for assembly and RSEM for TPM and count quantification followed by gene calling with prodigal. Two similar pipelines were used for CAZy and KO differing in annotation algorithm and database. Python scripts were used for data aggregation merging trinity ID, RSEM abundance and MEGAN taxonomy. KO and CAZy annotated transcripts were further calculated as transcripts * g^-1^ dw soil. The tailored KO approach selected genes relevant for the soil microbial carbon pump.

Figure S2A Barplots showing the mean abundance of Amoebozoa, Archaea, Bacteria, Fungi and Viruses for each treatment based on the non-assembled transcripts.

Figure S2B Barplots showing the mean abundance of bacterial phyla found in each treatment based on the non-assembled transcripts.

Figure S2C Barplots showing the mean abundance of archaeal phyla for each treatment based on the non-assembled transcripts.

Figure S2D Barplots showing the mean abundance of fungal phyla for each treatment based on the non-assembled transcripts.

Figure S3A Barplots showing the mean abundance of CAZy annotated transcripts on Level 1 for each treatment. AA = Auxiliary Activates, CE= Carbohydrate Esterase, GH = Glycoside Hydrolase, GT = Glycosyl Transferases, PL= Polysaccharide Lyases, CBM = Carbohydrate Binding Module.

Figure S3B Barplots showing the mean abundance of the five most abundant CAZy annotated families on level 2. Other CAZy families are summarized in other.

**Figure S4A** Principal Coordinates Analysis (PCoA) of CAZy-annotated transcripts based on transcripts * g^-1^ dw soil. Variance explained by PCoA1 and PCoA2 is shown on the respective axis.

**Figure S4B** Principal Coordinates Analysis (PCoA) of all KO-annotated transcripts based on transcripts * g^-1^ dw soil. Variance explained by PCoA1 and PCoA2 is shown on the respective axis.

**Figure S4C** Principal Coordinates Analysis (PCoA) of tailored analysis of KO-annotated transcripts based on transcripts * g^-1^ dw soil. Variance explained by PCoA1 and PCoA2 is shown on the respective axis.

**Figure S4D** Principal Coordinates Analysis (PCoA) of tailored analysis of KO-annotated transcripts based on TPM. Variance explained by PCoA1 and PCoA2 is shown on the respective axis.

Figure S5A KO-annotated transcripts of the anabolic category of the tailored KO analysis by growth stage (A) and admixture (B). Bubble size indicate mean values of transcripts * g dw soil without transformation. Data was log2(+1) transformed before two-way ANOVA with FDR p-value correction (* p < 0.05, ** p < 0.01 ***p< 0.001).

Figure S5B KO-annotated transcripts of the catabolic category of the tailored KO analysis by growth stage (A) and admixture (B). Bubble size indicates mean values of transcripts * g dw soil without transformation. Data was log2(+1) transformed before two-way ANOVA with FDR p-value correction (* p < 0.05, ** p < 0.01 ***p< 0.001).

Figure S5C KO-annotated transcripts the bacterial chemotaxis category of the tailored KO analysis by growth stage (A) and admixture (B). Bubble size indicates mean values of transcripts * g dw soil without transformation. Data was log2(+1) transformed before two-way ANOVA with FDR p-value correction (* p < 0.05, ** p < 0.01 ***p< 0.001).

Figure S5D KO-annotated transcripts of the Extracellular Polymeric Substance (EPS) category of the tailored KO analysis by growth stage (A) and admixture (B). Bubble size indicates mean values of transcripts * g dw soil without transformation. Data was log2(+1) transformed before two-way ANOVA with FDR p-value correction (* p < 0.05, ** p < 0.01 ***p< 0.001).

Figure 5E KO-annotated transcripts of the bacterial ABC transporter category of the tailored KO analysis by growth stage (A) and admixture (B). Bubble size indicates mean values of transcripts * g dw soil without transformation. Data was log2(+1) transformed before two-way ANOVA with FDR p-value correction (* p < 0.05, ** p < 0.01 ***p< 0.001).
